# Assembly collapsing versus heterozygosity oversizing: detection of homokaryotic and heterokaryotic Laccaria trichodermophora strains by hybrid genome assembly

**DOI:** 10.1099/mgen.0.001218

**Published:** 2024-03-26

**Authors:** Rodolfo Enrique Ángeles-Argáiz, Luis Fernando Lozano Aguirre-Beltrán, Diana Hernández-Oaxaca, Christian Quintero-Corrales, Mauricio A. Trujillo-Roldán, Santiago Castillo-Ramírez, Roberto Garibay-Orijel

**Affiliations:** 1 Posgrado en Ciencias Biológicas, Universidad Nacional Autónoma de México, Circuito de los Posgrados s/n, Ciudad Universitaria, Delegación Coyoacán, Ciudad de México, México, C.P. 04510, Mexico; 2 Instituto de Biología, Universidad Nacional Autónoma de México, Tercer Circuito s/n, Ciudad Universitaria, Delegación Coyoacán, Ciudad de México, México, C.P. 04510, Mexico; 3 Red de Manejo Biotecnológico de Recursos, Instituto de Ecología A. C. Carretera antigua a Coatepec 351, Col. El Haya, Xalapa, Veracruz, México, C.P. 91612, Mexico; 4 Centro de Ciencias Genómicas, Universidad Nacional Autónoma de México, Avenida Universidad s/n, Universidad Autónoma del Estado de Morelos, Cuernavaca, Morelos, México, C.P. 62210, Mexico; 5 Red de Biodiversidad y Sistemática, Instituto de Ecología A. C. Carretera antigua a Coatepec 351, Col. El Haya, Xalapa, Veracruz, México, C.P. 91073, Mexico; 6 Instituto de Investigaciones Biomédicas, Universidad Nacional Autónoma de México, Tercer Circuito s/n, Ciudad Universitaria, Delegación Coyoacán, Ciudad de México, México, C.P. 04510, Mexico; 7 Centro de Nanociencias y Nanotecnología, Universidad Nacional Autónoma de México, Km 107 carretera Tijuana-Ensenada, Ensenada, Baja California, Mexico, C.P. 22860, Mexico

**Keywords:** biotrophic fungal genome, hybrid genome assembly, intraspecific genome variability, NGS comparison, heterokaryotic fungal genome

## Abstract

Genome assembly and annotation using short-paired reads is challenging for eukaryotic organisms due to their large size, variable ploidy and large number of repetitive elements. However, the use of single-molecule long reads improves assembly quality (completeness and contiguity), but haplotype duplications still pose assembly challenges. To address the effect of read length on genome assembly quality, gene prediction and annotation, we compared genome assemblers and sequencing technologies with four strains of the ectomycorrhizal fungus *Laccaria trichodermophora*. By analysing the predicted repertoire of carbohydrate enzymes, we investigated the effects of assembly quality on functional inferences. Libraries were generated using three different sequencing platforms (Illumina Next-Seq, Mi-Seq and PacBio Sequel), and genomes were assembled using single and hybrid assemblies/libraries. Long reads or hybrid assemby resolved the collapsing of repeated regions, but the nuclear heterozygous versions remained unresolved. In dikaryotic fungi, each cell includes two nuclei and each nucleus has differences not only in allelic gene version but also in gene composition and synteny. These heterokaryotic cells produce fragmentation and size overestimation of the genome assembly of each nucleus. Hybrid assembly revealed a wider functional diversity of genomes. Here, several predicted oxidizing activities on glycosyl residues of oligosaccharides and several chitooligosaccharide acetylase activities would have passed unnoticed in short-read assemblies. Also, the size and fragmentation of the genome assembly, in combination with heterozygosity analysis, allowed us to distinguish homokaryotic and heterokaryotic strains isolated from *L. trichodermophora* fruit bodies.

**Impact Statement**

The sequencing of genomes here adds a third species to the collection of genomes of *Laccaria*, a model fungal genus for the physiology and ecology of ectomycorrhizal symbiosis. Three out of four assembled genomes were heterokaryotic dikaryons and one homokaryon. Unlike the available genomes of *Laccaria* sequenced from primary mycelium (n), here we sequenced secondary mycelium (assumed *n*+n) isolated from the context of wild fruit bodies. This is relevant given the genetic diversity present in the secondary mycelium, which is assumed to always contain two different nuclei with two parental origins. The sequencing of heterokaryon strains is a bioinformatics challenge, and the assembly procedures used here highlight a successful strategy for heterokaryon genome assembly. The detection of a non-heterokaryotic strain isolated from a fruit body opens the possibility of a homothallic life cycle in which a homokaryotic strain does not need to fuse its mycelium with a compatible one. This challenges the general idea of the heterothallic life cycle of Agaricales, although it has also been recently reported in some *Amanita phalloides* fruit bodies.

## Data Summary

This Whole Genome Shotgun project has been deposited at DDBJ/ENA/GenBank under accessions JACTVB000000000, JACTVC000000000, JACTVD000000000 and JACTVE000000000. The assemblies are available under accession numbers ASM1841795v1, ASM1841796v1, ASM1842394v1 and ASM1842387v1 and GenBank numbers GCA_018417955.1, GCA_018417965.1, GCA_018423945.1 and GCA_018423875.1. The BioProject and BioSample accessions are PRJNA642675 and SAMN15395802, SAMN15395803, SAMN15395804, SAMN15395805, SAMN39462802 and SAMN39462803. The sequencing libraries are available in SRA under accession numbers SRR13729862, SRR13729861, SRR13729860, SRR13729859, SRR13729858, SRR13729857, SRR13729856, SRR13729855, SRR27584511 and SRR27584512. Code and whole pipeline for the genome assembly assays and CAZyme annotation are available online (https://github.com/Rodolfo47/LtG.git).

## Introduction

From a functional genomic point of view, one of the most relevant characteristics of fungi within the order Agaricales (Dikarya, Basidiomycota) is that their life cycle is dominated by their dikaryotic stage, which refers to the two nuclei within each hyphal cell [1,3]. Most Agaricales, such as those of the genus *Laccaria*, begin their life cycle as monokaryotic and haploid basidiospores (n), the product of sexual recombination. These spores germinate to form primary mycelium, also monokaryotic and haploid (n), which fuses with another primary mycelium through somatic plasmogamy. The new fused hyphae have two different nuclei, one from each parental hypha. Both nuclei remain independent, forming the dikaryon (secondary mycelium) (*n*+n). Since each nucleus comes from a different parental primary mycelium, each has a different genome, which is why it is considered a heterokaryon. The dikaryon, which contains two genomes in independent nuclei, is responsible for most of the biological activities, such as vegetative growth, soil exploration, ectomycorrhizal symbiosis and basidiomata production for sexual sporulation [4]. Basidiomes are almost entirely formed by secondary mycelium (dikaryotic), except for particular cells in the superficial layer of the gills, the hymenium. These cells are the basidia, responsible for the production of basidiospores. Karyogamy occurs in immature basidia, in which the two different nuclei fuse for sexual recombination (2n). Subsequently, meiosis and post-meiotic mitosis immediately multiply the short-lived diploid nucleus to four haploid nuclei during basidia maturation. Each haploid nuclei migrates to one of the four basidiospores (n) produced by tetrasporic basidia (Fig. S1, available in the online version of this article) [5,7].

Fungal genome assembly accuracy relies on the interaction of three main factors: (1) intrinsic genome features such as those described above, (2) sequencing data and (3) computational approaches [8]. Each high-throughput sequencing technology has its limitations. For instance, in 454 pyrosequencing and Ion Torrent detection the correct length of homopolymeric regions is a major concern; the short paired-end reads from Illumina yield assemblies with a large number of contigs; and in massive long-read sequencing technologies, NanoPore produces the longest reads and PacBio resolves the error rate after several sequencing loops, so their assemblies are often less fragmented and more contiguous [9,11]. Nevertheless, overcoming the heterozygosity of non-haploid genomes remains problematic, potentially due to intrinsic genome variants between nuclear versions. However, to offer an accurate haploid genome, there are several assembly approaches such as *k*-mer sorting, graph construction, aligning and overlapping of short or long reads, scaffolding, filling gaps, correcting misassembly, polishing results and purging for haplotypes [12,16].

It has been estimated that the genome size of ectomycorrhizal fungi, such as *Amanita* species, could be larger than those produced by short-read sequencing data due to the large number of repeated elements [17,18]. This phenomenon is promoted by particular families of transposable elements [17,18] and could be prominent on large genomes of plant-associated biotrophic fungi [19], for example ectomycorrhizal fungi [20]. Assembly of large and highly repetitive eukaryotic genomes has been addressed using third-generation sequencing technologies and improving computational tools (e.g. [21,24]). Recently, resolved genome assemblies of nuclei variants of heterokaryotic fungi in *Lentinula* have been reached by combining long and short reads with optical mapping posterior of a protoplast dedikaryotization. This involves enzymatically eliminating the cell wall, propagating the protoplasts and, after cell wall regeneration, detecting monokaryons by mating colonies with non-clamped hyphae, so sequencing was done from synthetic monokaryons that originated from an original heterokaryotic dikaryon [25]. Recently, dikaryotic homokaryotic genomes were detected in wild basidiomata of *Amanita phalloides* [26].

To understand the effects of read length on the quality of *de novo* genome assembly of an ectomycorrhizal fungus and its effect on functional inferences, we selected four strains of *Laccaria trichodermophora* Muell. 1984 for wide genome shotgun sequencing. All strains were isolated from fresh basidiomata, so they were likely to be composed of heterokaryotic dikaryotic mycelium. We measure the quality of a genomic assembly by evaluating its completeness and contiguity. The quality of the assembly reflects the accuracy of the genomic hypothesis. Additionally, our analyses highlight strain differential heterozygosity.

## Methods

### Strain origin and derivation

*L. trichodermophora* was selected due to its cultural and biotechnological importance [27,30]. This species is a close relative of *Laccaria bicolor* (Maire) Orton 1960, the genome of which was the first of a mycorrhizal fungus sequenced by cloning in plasmids and fosmids from the monokaryon strain S238N-H82 [31]. These two lineages diverged around six million years ago [32] and share macromorphological features [33]. A second genome from *Laccaria*, that of *Laccaria amethystina* LaAM-08-1, was obtained with paired-end second-generation sequencing technology, also from a monokaryotic strain [34]. *L. bicolor* and *L. amethystina* are host generalists and widely distributed; in contrast, *L. trichodermophora* is restricted to Northern and Central America, and has a strong preference for pine hosts [27,35,44]. However, it can shift hosts when outside of its natural distribution [45,47].

We used previously generated population genomics, and phenotype data for strain selection (Table 1). Ángeles-Argáiz *et al*. [27] reported the field sampling and strain isolation from dikaryotic basidiomata of several *L. trichodermophora* samples and their culture kinetics. Furthermore, Quintero-Corrales *et al*. [43] found that the genetic diversity and structure in *L. trichodermophora* populations across the Transmexican Volcanic Belt was determined by geographical distance. The selection of the four strains used here was designed to represent the wide intraspecific (genetic and phenotypic) diversity of the species.

**Table 1.** Strain derivation and accessibility

Strain	CA15-11	CA15-75	CA15-F10	EF-36
Collection	CA15-11	CA15-75	CA15-F10	REAA13-59
MEXU voucher	29 986	29 985	29 987	27 575
XAL strain	IE-6004	–	IE-6010	IE-6012
Origin	SN	LM	nt	VP
Latitude (°N)	18.98	19.28	19.12	19.12
Longitude (°W)	−97.29	−98.04	−99.77	−99.21
Elevation (m asl)	3836	3067	4028	3094
Host	*Pinus* hartwegii	*Pinus montezumae*	*Pinus hartwegii*	*Pinus montezumae*
NCBI ITS	MN710460	MN710467	MN710481	KT354980
NCBI genome assembly	GCA_018417965.1	GCA_018423945.1	GCA_018423875.1	GCA_018417955.1*

*Reference genome. NCBI ITS = GenBank Internal Transcriber Spacer sequence number.

LM La Malinche, Tlaxcala
nt
Nevado de Toluca, Mexico Estate SN Serra Negra, Puebla VP Volcán Pelado, Mexico City

All four strains used here were isolated from the context of fresh basidiomata, and they are considered to be dikaryotic (secondary mycelium). In a flow hood, using cold flamed surgical knives and tweezers, clean tissues from the interior of the stipe base were placed on modified melin-norkrans (MMN) plus chloramphenicol agar plates. Growing mycelia were reseeded on potato dextrose agar (PDA) until pure cultures were obtained. The strains were stored at 4 °C on PDA and reseeded for maintenance.

### Sequencing method and library features

DNA extractions were performed from 16-day-old cultured secondary mycelium of the four strains. The mycelial cultures were grown in 250 ml baffled shake Erlenmeyer flasks filled to 20 % (50 ml of modified BAF culture medium, at 25±2 °C, 100 r.p.m. and initial pH of 5.5). Mycelial cultures were filtered through sterile Waltman #4 filters using a vacuum plump. Filtered mycelia were stored for a few days in sterile Falcon tubes at −70 °C until downstream procedures. DNA extractions of high purity and integrity were achieved using the DNeasy Plant Mini Kit (Qiagen) with modifications (Method S1). Before genomic sequencing, extracted DNA quantity was measured with NanoDrop v4 and Qubit v1, and purity was determined with NanoDrop.

For each strain, two short-read (SR) Illumina libraries were sequenced in the ‘Instituto Nacional de Medicina Genómica’ in Mexico City, and for two strains, PacBio long-read (LR) libraries were sequenced by MacroGen in South Korea (Table 2; Figs S2–S9). One of the SR libraries was produced using four Illumina Next-Seq flow cells per sample with 2×76 bp with overlap. The second SR library was made with a full Illumina Mi-Seq flow cell per sample with 2×300 bp with an average distance of 100 bp between forward and reverse reads. For LR libraries, one full PacBio Sequel 1 flow cell per sample was used to obtain libraries mainly >10 kb in read-length. Sequel’s Circular Consensus Sequencing provides a low error rate in base assignment, and although it is not the technology that generates the longest sequences, it produced reads >90 kb in length. For PacBio sequencing we selected the strain with the greatest amount of physiological data (EF-36) and a geographically distant one (CA15-11).

**Table 2.** Shotgun whole genome sequencing data summary

Strain	Illumina Next-Seq (2×76 bp) (SR)			Illumina Mi-Seq (2×300 bp) (SR)			PacBio Sequel 1 (>10 kb) (LR)			
	Raw reads	Clean reads	Mean coverage	Raw reads	Clean reads	Mean coverage	Subreads	Reads	Corrected reads	Median coverage
CA15-11	106 996 984	63 107 798	40	4 066 964	4 022 332	21	1 293 905	679 396	191 573	148
CA15-75	106 996 984	103 956 030	166	6 797 912	6 634 218	42	–	–	–	–
CA15-F10	64 249 826	26 371 613	82	2 040 731	2 019 889	12	–	–	–	–
EF-36	128 404 683	124 772 395	319	7 981 238	7 787 611	80	1 038 541	709 445	181 727	136

### Genome assembly

Several *de novo* assembly assays were performed using different assembler algorithms for each of the three libraries alone or combined. Bioinformatic procedures can be summarized in three main steps: (1) cleaning, (2) assembling and (3) refining. A detailed schematic workflow is shown in Fig. S10.

SR data cleaning was performed with Trimm-Galore v0.6.4 (default phred score: 20) [48] and the quality controls of raw and clean data were assessed using FastQC v0.11.8 [49] (Figs S2–S9). For paired-end assembler selection we started by using only one strain (EF-36) and one clean library (2×76 bp). The first step was detecting a working *k*-mer size via Velvet-Optimizer v2.2.6 [50], which resulted in *k*=71. All assembler tests thus used *k* values around 71. ABySS assembler v2.0.1 [51] was tested with 53, 63, 73, 83 and 93 *k*-mer sizes. IDBA v1.1.3 [52] was used with interleaved reads; the *k*-mer size was between 31 and 121 with 10 bp steps. SPAdes v3.13.1 [53] was used with three *k*-mer size combinations: *k*-mer size ranged between 31 and 71 with steps of 10 bp, *k*-mer size ranged between 31 and 75 with steps of 4 and 6 bp, and with default parameters. The *k*-mer sizes tested with Velvet v1.2.10 [50] were 67, 69, 71, 73 and 75. Although these four assemblers are based on *k*-merisasion and construction of De Bruijn graphs, Velvet is more efficient with small genomes such as of bacteria; ABySS was designed to handle large data sets; IDBA creates the graphs iteratively, which is why it is considered effective for resolving difficult regions in the genome; while SPAdes includes steps after construction of the graphs to increase the contiguity of the assembly.

We performed quality assessment of all assembled genomes with QUAST v5.0.2 [54]. The choice of the ‘best’ result of each assembler was done seeking for maximum contiguity, following the criterion: low contig number and L50 values, but high N50 and contig size values. The remaining strains were assembled following the parameters that gave the best outcomes. Those assemblies were used for scaffolding by SSPACE v3.0 (‘SSPACE_Standard’ [55]), and processed with GapFiller v1.10 [56] in order to obtain a highly accurate genome assembly with SR.

LR assemblies were made with Canu v2.0 [57] on diploid and smashing haplotype modes, and with Fly v2.8.3-b1695 [21]. *De novo* hybrid assembly approaches vary in the use of the input data and could be classified into three groups: (1) methods that start assembling SRs and then LRs to generate longer contigs; (2) methods that correct raw LRs using SRs and then build contigs with corrected LRs only; and (3) methods that assemble LRs first and then use SRs to polish the resulting assembly. For the first method group, we used hybridSPAdes v3.13.1 [58] to assemble SRs and then we used LRs to extend and join contigs. For the second method, we used MaSuRCA v3.3.8b [59] to correct raw LRs using SRs and then to build contigs using corrected LRs. For the third method, we built contigs with wtdbg2 v2.5 [60] using LRs and then polished them with the SRs. Also, a fourth method was tested where an assembly was made using self-corrected LRs (using Canu v2.0 in diploid mode), scaffold contigs with LRs (using SSPACE-LR v1.1 [61], then improving the resulting assembly by filling gaps with SRs (using GapFiller v1.10), and finally detecting mis-assemblies and indels or blocking substitution events with three rounds of the polishing tool Pilon v1.23 [62]. Pilon used the SRs and LRs mapped over the assembly with Bowtie2 v2.3.5 [63] and Minimap2 v2.17 [64], respectively. Reads were aligned to the polished previous round assembly for the second and third Pilon polishing rounds. From the polished diploid assemblies, diploid repeated contigs were detected and discarded using the Purge Haplotigs pipeline v1.1.1 [65].

Genomic completeness and duplicate evaluation were made with BUSCO v4.0.5 [66] by comparing the genomic assemblies versus the Agaricales database (agaricales_odb10). Sequencing coverage was estimated with Minimap2 v2.17 and BBtools pileup.sh [67]. QUAST was also used to obtain general assembly quality features.

Finally, representative assembly results were selected. Single (76 and 300 bp) and both SR assemblies of the four strains were obtained from SPAdes plus scaffolding and gap filling. Furthermore, the Canu assemblies (only LR) and the Canu plus polishing (hybrid assemblies) for the two strains with LRs were selected. These three (CA15-F10 and CA15-75) or five (CA15-11 and EF-36) genome assemblies per strain were used for annotation and subsequent analyses.

### Non-targeted organism identification in LR assemblies

Due to the large differences found between the assemblies generated with short reads and long reads, and to identify and dismiss contaminations coming from non-targeted organisms, we analysed the genomic assemblies as if they were metagenomes using MaxBin v2.2.1 [68] and VizBin v1.0.0 [69]. MaxBin analysis uses a bacterial gene library (housekeeping and ribosomal) for taxonomic assignment of contigs within the assembly. VizBin generates a two-dimensional map of the contigs by performing a Barnes-Hut Stochastic Neighbor Embedding (BH-SNE) on the centred log ratio-transformed sequence signatures such as GC content and tetramers. From the VizBin result (scatterplot), dots far from the main dot-cloud were treated as outliers and considered as potential contaminants. The online version of blast [70] was used against the NCBI nr database for taxonomic assignment of contigs considered as potential contaminants.

### Genome size and heterozygosity calculation by *k*-mer coverage

Jellyfish v2.3.0 [71] was used to estimate the genome size of the samples without underestimation due to collapsed repeated regions within the SR assemblies or obtain an oversized assembly due to the heterozygosity recovered by the LR. The *k-*mer sizes (19, 21, 23, 25 and 27) were large enough to allow them to map uniquely to the genome. The haploid genome *k*-mer frequency must follow a Poisson distribution around a single peak of the mean coverage of *k*-mer counts [72,73]. Heterozygosity values and genome size were calculated using the GenomeScope online tool [74].

### Mating type locus analyses

As a complementary analysis to identify the heterozygosity of the strains, we looked for mating-type loci. The amino acid sequences of the HD1 (XM_001873350) and STE3-like genes (XM_001882453.1, XM_001886543.1, XM_001888530.1, XM_001888531.1, XM_001888575.1 and XM_001888577.1) of *L. bicolor* were used as queries to find these genes in the genomes of *L. trichodermophora* with tblastn [75]. Subsequently, a previously characterized region of *L. bicolor* [76] (around 100 kb) was extracted and used to extract the *L. trichodermophora* contigs that matched the region. The contigs (of hybrid assemblies), of the EF-36 and CA15-11 samples where the MAT-A region was found, were compared via MUMmer v4 [77] in Geneious Prime v2022.2.2 to identify their synteny.

### CAZyme annotation and comparative analysis

Carbohydrate activity enzymes (CAZymes) are a reduced but important gene set on the ectomycorrhizal symbioses [20,34, 78]. Thus, to identify the effect of the assembly quality on the functional inferences, we focused on the analyses of these enzymes. For each strain, all assemblies were used in the gene model prediction with AUGUSTUS v3.2.3 [79] trained with the *L. bicolor* cDNA (--species=laccaria_bicolor), in single strand and complete gene model mode, allowing non-alternative transcripts. CAZyme coding genes were annotated with dbCAN2 v2.0.11 [80]. Only HMMER [81] outcomes from the CAZy database [82] were considered for comparative analyses [83].

## Results and discussion

### Genome assembly

Based on the high contiguity and completeness of the assemblies, the SR SPAdes assemblies were selected for further refinement; the Canu LR assembly was selected; and as hybrid assemblers the Canu plus Pilon assemblies were selected (Tables S1–S6). Sixteen *L. trichodermophora* genome assemblies, representing the genomes of the four strains, were subjected to annotation and further comparison (Table 3). All strains have SR data but just two (CA15-11 and EF-36) have LR data.

**Table 3.** *Laccaria trichodermophora* genome features and assembly comparison

Assembly	76 bp	300 bp	SRs	LRs	Hybrid
**Strain CA15-11**					
Total length (Mb)	46.86	68.15	70.78	111.69	**111.90**
Contigs	14 968	20 119	18 965	1119	**876**
Largest contig (kb)	55.40	86.95	86.72	917.15	1052.63
N50 (kb)	4.11	4.70	5.49	171.95	**241.04**
L50	3172	3985	3456	180	**133**
Gene number	23 114	31 420	31 734	34 575	34 713
Genome completeness (%)	50.8	60.6	61.4	94.2	**95.7**
Genome duplication (**%**)	**1.7**	7.0	8.7	30.5	37.6
Exome length (Mb)	22.16	30.30	31.76	50.13	51.09
Genetic density (**%**)	47.30	44.46	44.87	44.89	45.66
CAZymes	243	349	363	525	**537**
GC (%)	47.47	47.13	47.12	46.65	46.67
No. of N’s per 100 kb	144.59	91.10	103.71	**0.00**	349.37
**Strain CA15-75**					
Total length (Mb)	46.72	66.75	**75.35**	–	–
Contigs	13 822	18 038	18 845	–	–
Largest contig (kb)	125.60	126.77	**127.70**	–	–
N50 (kb)	4.65	5.55	**6.09**	–	–
L50	2820	3239	3292	–	–
Gene number	22 384	29 941	31 229	–	–
Genome completeness (**%**)	48.10	57.80	**65.10**	–	–
Genome duplication (**%**)	**2.70**	9.40	15.20	–	–
Exome length (Mb)	22.15	29.95	33.35	–	–
Genetic density (**%**)	47.41	44.87	44.26	–	–
CAZymes	250	358	**392**	–	–
GC (**%**)	47.43	47.07	47.04	–	–
No. of N’s per 100 kb	184.43	**100.82**	152.65	–	–
**Strain CA15-F10**					
Total length (Mb)	41.23	57.29	**65.27**	–	–
Contigs	15 893	19 562	18 694	–	–
Largest contig (kb)	83.35	91.45	**91.72**	–	–
N50 (kb)	3.03	3.76	**4.94**	–	–
L50	3718	4196	3603	–	–
Gene number	21 334	27 242	29 663	–	–
Genome completeness (**%**)	46.2	65.3	**64.7**	–	–
Genome duplication (**%**)	**0.5**	2.3	6.7	–	–
Exome length (Mb)	19.32	25.70	29.54	–	–
Genetic density (**%**)	46.84	44.86	45.27	–	–
CAZymes	221	310	**347**	–	–
GC (**%**)	47.60	47.29	47.21	–	–
No. of N’s per 100 kb	386.15	168.65	**114.08**	–	–
**Strain EF-36**					
Total length (Mb)	43.82	50.32	50.33	58.99	**59.06**
Contigs	3787	3434	3341	94	**77**
Largest contig (kb)	504.87	568.43	576.99	4959.16	5316.68
N50 (kb)	29.86	43.00	43.96	1351.60	1464.17
L50	363	299	283	**12**	**12**
Gene number	16 168	17 522	17 579	18 691	18 708
Genome completeness (**%**)	97.4	97.3	97.5	97.5	**97.6**
Genome duplication (**%**)	**1.0**	1.3	1.2	1.6	1.6
Exome length (Mb)	22.91	25.03	25.10	28.38	28.47
Genetic density (**%**)	52.28	49.74	49.87	48.11	48.20
CAZymes	309	318	318	**322**	320
GC (**%**)	47.70	47.23	47.24	46.84	46.84
No. of N’s per 100 kb	9.40	37.96	26.55	**0.00**	91.36

Bold values denote best assembly parameters.

**Fig. 1.**
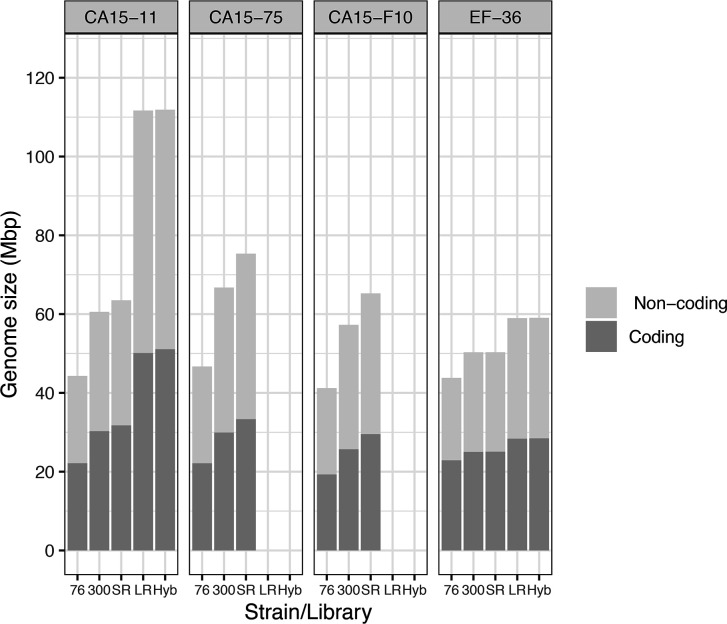
Effect of strain and assembly method on *L. trichodermophora* genome size and gene density. 76=SPAdes assembly with Next-Seq library of 2×76 bp, 300=SPAdes assembly with Mi-Seq library of 2×300 bp reads, SR=Refined assembly with both short read libraries, LR=Canu assembly with Sequel long-read library, and Hyb=Hybrid polished Canu+Pilon assembly with LR and both SR libraries.

By comparing assembly size and genetic content of the three types of assemblies that use just SRs (76 bp, 300 bp and SRs) for each of the four strains, it was evident that read length does influence assembly size. As read length increases so does the genome assembly length (correlation values: tau=0.61, *P*=0.003; rho=0.73, *P*=0.001, Figs 1 and S11). Considering only contigs larger than 1 kb, assemblies of 2×76 bp read length (76 bp) of strains CA15-11, CA15-75, CA15-F10 and EF-36 resulted in genome sizes of ~47, 47, 41 and 44 Mb, respectively. With this read length the EF-36 genome was the best assembled, with 3787 contigs and N50 of 29.86 kb, compared to 13 822–15 893 contigs of the other genomes. With 2×300 bp read length, all genomes increased their sizes to ~68, 67, 57 and 50 Mb. The genome of EF-36 was the smallest but the best assembled, with 3434 contigs compared to 18 038–20 119 contigs of the other strains. It is of note that the genome of strain EF-36 (homokaryon) decreased in contig number but increased in genome size, while the remaining three strains (heterokaryon) increased in genome size and also in contig number. Using both combined SR libraries (2×76/300 bp) genome size continued to increase (~71, 75 and 65 Mb) (except in EF-36: ~50 Mb), but the contig number decreased (except in CA15-75). For all strains, as read length increased, the number of predicted genes also increased (Table 3).

For the SR libraries, the CA15-75 genome was the largest, with 75.35 Mb and 31 229 predicted genes, while EF-36 was the smallest, with 50.32 Mb and 17 579 predicted genes. Simultaneously, the smaller genome strain (EF-36) was the one with lower fragmentation (∼3000 contigs vs. almost 19000 contigs). Also, EF-36 showed less variation in genome size and gene density compared with the 76 bp, 300 bp or SR assemblies (Table 3, Fig. 1). Notwithstanding this, only LR libraries could cover specific genomic regions (Figs S12 and S13).

Comparing the earliest genomes generated by Sanger sequencing (no sequencing depth with long reads) against genome assemblies using short reads (great sequencing depth but heterogeneously distributed across the genome), it is clear that longer reads are better than a high sequencing depth when genome architecture and completeness are the objectives [84]. Therefore, hybrid approaches combining high-depth SR libraries with LRs (Sanger, 454, Illumina Mate-Pair, and more recently PacBio and NanoPore) have shown better results (e.g. [24,85]). In our data, it was evident that LR assemblies were larger than SR assemblies regardless of strain identity. Also, we found differences among strain genome lengths. Long reads increased the contiguity of assemblies (CA15-11 from 18 965 SR contigs to 1119 LR contigs; EF-36 from 3341 SR contigs to 94 LR contigs). The increase in genome size of the strain with the largest and most fragmented assembly (CA15-11) was more prominent than the smallest and most well-solved one (EF-36) (~58 vs. 17 %, respectively) (Fig. 1).

Because there were huge differences between SR and LR assemblies, we used metagenomic approaches to determine if genomic DNA contamination in LR libraries was the cause. No bin was assigned to bacterial taxa (Table S7) using MaxBin. The potential outlier contigs obtained with VizBin analyses match genes or hypothetical proteins of available genomes of *L. amethystina* and *L. bicolor* in NCBI (Fig. S14; Table S8).

Notwithstanding the low fragmentation of the LR assemblies and despite the reads self-correction by the Sequel circular consensus sequencing, hybrid assembly takes advantage of the accurate base calling and high coverage of the short-paired reads to break potential misassemblies and improve base assignation [86]. Here, to obtain a larger size of the assembly with less fragmentation, the Canu+Pilon method offered the best result (Table S6). In hybrid assemblies, genome lengths were similar to those of LR assemblies but worked better in terms of fragmentation and gene prediction.

Fungal genomes are very dynamic in nature. Even members of the same genus can show remarkable divergence at the genomic level, in terms of amino acid sequence identity and genome synteny [87] . Using a hybrid approach, Tobias *et al*. [24] completed a high-accuracy assembly of the early divergent Basidiomycete rust *Austropuccinia psidii* (1018 Mb), while previous draft assemblies of this fungus estimated genome size an order of magnitude less, between 103 and 145 Mb [88]. However, that rust (Pucciniomycotina) is distantly related to *Laccaria* (Agaricomycotina) and belongs to a clade in which several phytopathogens have evolved very large genomes [89]. It has been suggested that ectomycorrhizal fungal genomes in Agaricales such as *Amanita* are 8 % to >600 % larger than its current assemblies. The huge size of some *Amanita* genomes was attributed to expansions of repeat regions, populated by transposable elements, but poor in coding genes [17,18]. Here, the *L. trichodermophora* CA15-11 assembly reached near double the size of the EF-36 assembly and also had a twofold higher gene account, so the non-coding DNA was not the cause of the increase.

**Fig. 2.**
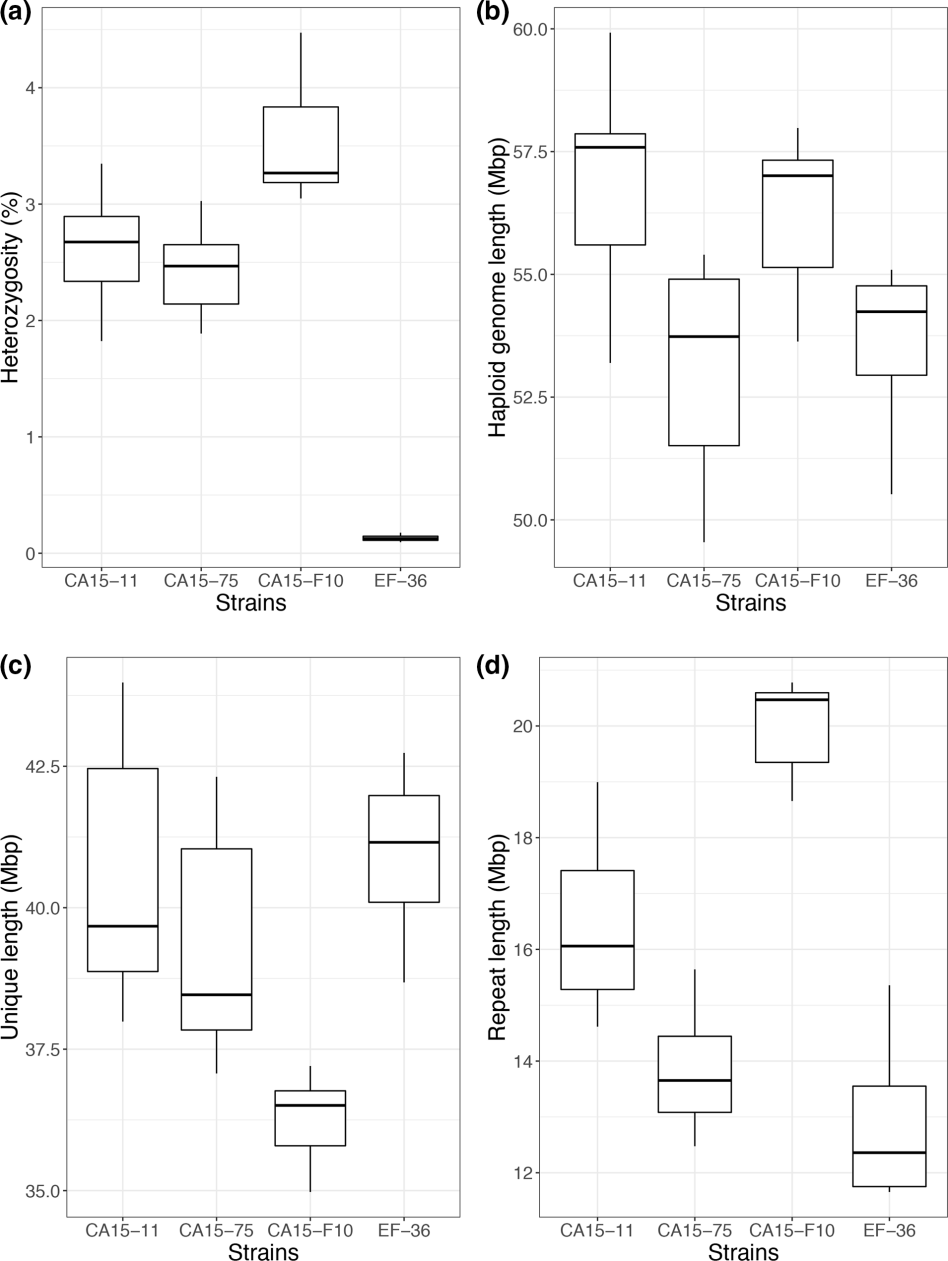
Heterozygosity and genome sizes predicted by *k-*mer coverage using Jellifish and GenomeScope. (**a**) Heterozygosity values, (**b**) haploid genome size estimation, (**c**) unique genome length and (**d**) duplicated genome length.

The *L. trichodermophora* genomic assemblies generated with SRs were also smaller than estimated by *k*-mer coverage. These size estimations resemble the strain EF-36 genome assembly size but are half the strain CA15-11 assembly. The Jelifish-GenomeScope analysis also presented *k-*mer count values that indicate three of the four strains as heterokaryons but EF-36 as a monokaryon (Figs 2 and S15).

Revision of the diploid genomes of both strains with Purge Haplotigs had a minor impact on their primary assemblies. Only ten secondary contigs were reassigned as allelic contigs and dismissed from the primary assembly of strain CA15-11 (<281 kb, 0.25 %), and no contig of strain EF-36 was reassigned. Finally, hybrid assemblies of CA15-11 reached 111.90 Mb genome length while EF-36 reached 59.06 Mb, parcelling into 876 and 77 contigs respectively, including nuclear and mitochondrial genomes.

The assembly size of the strain with the largest and most fragmented SR assemblies (CA15-11) was about double that of the genome of the smallest and less fragmented one (EF-36). The structural variations given by the meso-synteny could cause contig identity values to be not sufficiently high, impeding Purge Haplotigs analysis from eliminating haplotypes.

The *L. trichodermophora* EF-36 genome assembled with the hybrid approach was around half the length compared with strain CA15-11 (Fig. 1). Although often deleterious, evidence of large-scale genomic profit/loss in both overall ploidy/heterozygosity and individual chromosomal copy numbers has been reported in yeasts, with biotrophic and saprotrophic lifestyles [90,93], sometimes attributed to laboratory maintenance [94]. In contrast to evidence for genomic expansion–reduction, genome assembly of the model plant *Arabidopsis thaliana*, performed by Canu, was over-sized (196.5 Mb) compared to the expected size of 144 Mb, due to its high heterozygosity that led to artefactual locus multiplications [9]. Similar heterozygosity-mediated overestimation may have occurred with *L. trichodermophora* genome assemblies, if using a genome concept as the representation of a single haploid version of it.

The size of the genome assemblies of six *Rhizophagus irregularis* (Glomerales, Mucoromycota) conspecific isolates ranged from 122 to 138 Mb, with large numbers of orphan genes and strain-specific gene family expansions [95]. Unlike most Agaricales, more than one nucleus is found in a single spore. The genomes of several individual nuclei in the same spore were analysed by flow cytometry and single cell sequencing. The authors found differences in genome size, organization and genetic content [96]. Nevertheless, haplotype-resolved genomes of *Lentinula edodes* (Agaricales, Basidiomycota), achieved by protoplast dedikaryotization, next-generation sequencing (NGS) and optical mapping, showed slight differences in genome size, but strong differences in the content and genetic organization of each nucleus [25]. The content and genetic organization discordance of the two nuclei in *L. trichodermophora* strains may explain the assembly fragmentation and size differences.

Recently, two homozygous fruiting body-forming fungal genets (individuals) were detected in a sample of 37 *A*. *phalloides* genets. These two genets were detected by NGS of several fruiting bodies for each one, some being found in different years in the same area [26]. To identify the heterozygosity of all *A. phalloides* genets, the authors used a similar analysis to ours. As with *L. trichodermophora* strain EF-36, the two homozygous *A. phalloides* genets showed a single peak in the *k-*mer depth analysis, while the other 35 *A*. *phalloides* genets showed the same pattern as the three remaining *L. trichodermophora* strains, with a major and a minor peak in *k-*mer depth. Here, haploid genome sizes of all strains were predicted around 55 Mb. These sizes resemble the shorter and potentially haploid genome assembly (EF-36) (Fig. 1), so the large assembly size must be attributed to the dikaryotic heterokaryotic nature of the secondary mycelia from where the strains were isolated. Our results indicated the presence of both heterokaryotic and homokaryotic *L. trichodermophora* strains in the laboratory, and raised the question of homokaryotic fruit bodies in the wild.

### Mating type loci

Search of the HD1 (MAT-A) gene of *L. bicolor* in the genomes of the *L. trichodermophora* strains gave two matches for all the three heterokaryotic strains and only one in the monokaryotic strain (Fig. S16, Table S8). No match was detected for the STE3-like (MAT-B) gene. A wide region around the MAT-A locus of *L. bicolor* aligned with that of EF-36 simply with a rearrangement, while for heterokaryotic strains high fragmentation and duplication were observed throughout the region (Fig. S17). Mauve analysis of the 100 kb region of the MAT-A locus of strains EF-36 and CA15-11 showed duplications equivalent to two-thirds of the region in the CA15-11 assembly with respect to EF-36 (Fig. S18). These findings point to opposing assembly phenomena: core regions, conserved in gene content and synteny between the pair of nuclei of each strain, are achieved by collapsing assembly; and rearrangements and complementary genes or alleles were assembled into independent contigs, causing fragmentation and (haploid) genomic size overestimation.

### CAZyme annotation comparative analysis

**Fig. 3.**
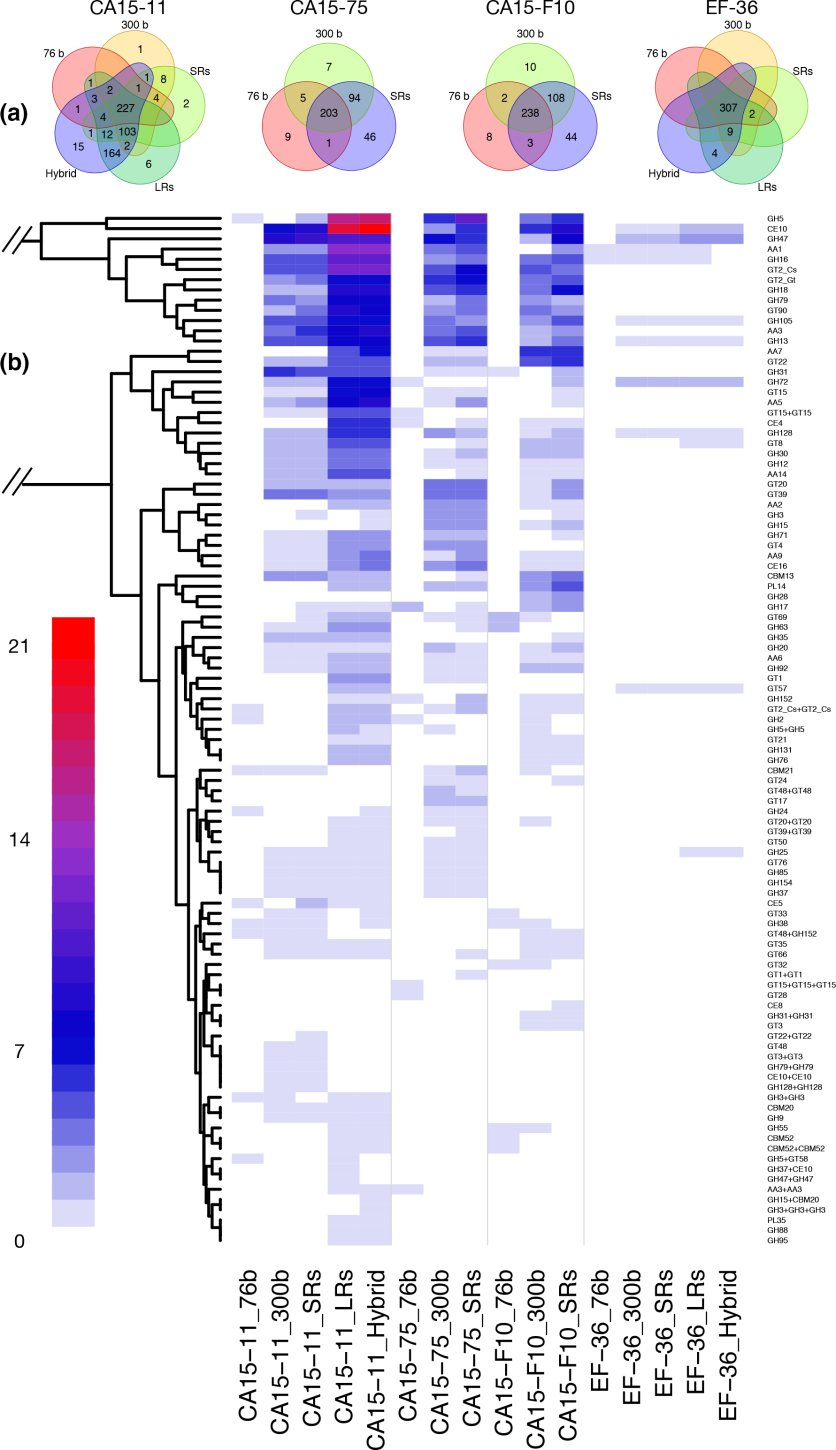
Comparison of predicted CAZyme content of four strains of *Laccaria trichodermophora* genomes assembled by three or five methods/libraries. (**a**) Venn diagram grouping the CAZymes common or exclusive for the different assemblies for each strain. (**b**) Heatmap comparing the predicted CAZyme gene content. Only differences are plotted. Genes common to all assemblies were not included in the graphic (for the full CAZyme set, see Fig. S20). White colour corresponds to no differences in number of genes; blue, purple and red indicate copy number additions as indicated in the colour key. Row dendrogram clusters together enzymes with similar copy number means.

Read length and assembly methods did influence genomic functional inferences. This phenomenon was reflected on the CAZyme gene set annotation (correlation values: tau=0.61494, *P*=0.003045; rho=0.7291531, *P*=0.001351; Fig. 3). In general, as the read length increased so did the number of CAZymes. However, losses of CAZyme genes at a lower proportion were also found (Fig. S19). Fig. 3(a) shows the shared and unique CAZyme genes to the three or five assemblies of each strain.

When comparing the CAZyme contents of the three assemblies generated with SRs, all strains increased in their gene numbers by almost a third using the 300 bp read length compared with the 76 bp read length. However, in the homokaryotic strain EF-36, the differences were just ~10 %. Using both SR libraries, few additional CAZymes were detected in the three heterokaryotic strains, but none in the homokaryotic strain EF-36 (Table 3, Fig. 3). In the well-resolved genome (EF-36), CAZyme number showed minimum variation with assembly approaches regarding read length increase (309, 318, 318, 322 and 320 CAZymes). In contrast, in the more complex genome (CA15-11), CAZymes more than doubled in number (243, 349, 363, 525 and 537 CAZymes). In most cases, the variation in the size of each CAZyme family ranged between one and seven copies (blue colours in Fig. 3b); there were outstanding cases, such as CE10 or GH5, where the variation was more than 15 copies (purple and red colours in Fig. 3b). Within the CE10 family, there were arylesterases and lipases involved in the metabolism of fatty acids and vitamins. The CE10 of *L. trichodermophora* was not assigned to specific subfamilies by dbCAN due to its rareness. Enzymes in GH5 are active on cellulose and hemicellulose. The GH5 enzymes annotated in the *L. trichodermophora* genomes accounted for at least seven sub-families, GH5_9 being the most affected by type of assembly. This means that when assembling heterokaryotic genomes with SRs, the glucanase, xylanase, glucosidase and mannosidase capacities were underestimated; in contrast, there was no variation for strain EF-36 in these enzymes.

Enzymes such as GT8 and GH25 in strain EF-36 could only be detected when assembling with LRs, while in strain CA15-11 this phenomenon was detected for more than 20 enzymes, in addition to several enzymes forming homomeric or heteromeric dimers that were only assembled as such by LRs. In extreme cases, such as AA7 or CE4, the use of LRs detected several copies, while the SRs did not detect any (Figs 3 and S20). Consequently, several oxidizing activities on glycosyl residues of oligosaccharides and several chitooligosaccharide acetylase activities would have passed unnoticed in SR assemblies.

### General conclusions

Comparison of assembly methods using different sequencing libraries and assemblers showed their effect on genome quality and gene prediction. Among the *L. trichodermophora* genomes analysed, we detected that one did not present the expected heterokaryotic nuclei pair for a strain obtained from a basidiome, but rather it showed a homokaryotic genomic pattern. This was confirmed by the presence of only one version of the mating-type locus HD1 gene in strain EF-36 and two in each of the other strains (CA15-11, CA15-75, CA15-F10). This discovery is relevant by itself since it is generally accepted that the fusion of two monokaryotic compatible mycelia and their development into a heterokaryotic secondary mycelium is essential to produce basidiomata and for the establishment of ectomycorrhizal symbiosis with plant roots [4,97]. *Laccaria fraterna*, a distantly related species, produces basidia that are typically bisporic (occasionally trisporic or monosporic), and the mycelium that arises from the germination of the spores, which are usually binucleate, have clamp connections, and two nuclei in most cases [98]. Fruiting bodies of *L. trichodermophora* from which the four strains were isolated have bisporic, trisporic and tetrasporic basidia and clamp connections (Fig. S21). Clamps are the mechanism by which dikaryotic hyphae transfer one of the two nuclei to the new cell during cell division. However, *Coprinopsis cinerea* and *Schizophyllum commune* are examples of fungi without clamps in their dikaryons if both nuclei present identical MAT-A alleles [99].

The presence of a single genomic set in strain EF-36 suggests a possible homothallic life cycle in which a homokaryotic strain does not need to fuse its mycelium with a compatible one. This challenges the general idea of the heterothallic life cycle of most *Laccaria* species. As the MAT-B gene was absent from this genome, it opens the question of a possible bipolar mating type. Classical mating genetics, flow cytometry and exhaustive spore counts per basidia could provide new insights into the life cycle of *L. trichodermophora* populations.

Sequencing and assembly also influenced gene prediction and CAZyme annotation, and this effect was different for heterokaryotic and for non-heterokaryotic strains. While the homokaryon assembly (EF-36) was better resolved by increasing the read length, the heterokaryotic assembly (CA15-11) continued to be highly fragmented. This heterokaryotic assembly is made up of the two different haplotypic nuclear versions, so it is duplicated. This could be considered a duplication artefact when using the concept that the genome is a haploid representation of the genetic information. However, in nature the cells of this strain do have both haplotype versions. When comparing the enzyme annotation, the increase in enzyme number is not simply explained by a doubling in number, as the heterokaryotic LR assembly (duplicate, CA15-11) also showed an increase in functional diversity. Although both nuclei share most of the genes, in general they present low synteny, and this phenomenon is responsible for the high fragmentation of the heterokaryotic assembly. Furthermore, as has been shown for other fungi [25], the two nuclear versions seem to share a genetic core, but also each present an accessory fraction. From a functional point of view, we consider core genes as duplicates, but accessory genes are not. Some of these accessory genes were only detected when using LRs.

Our results showed that read length impacts not only on the fragmentation rate or genomic size estimation but also on functional inferences, as illustrated in the CAZyme gene set. Genome underestimations can have serious effects on the detection of gene family diversity, causing misinterpretations in the strength of functions, which could be prominent in complex genomes of heterokaryotic fungi. Furthermore, when gathering evidence from different sequencing libraries, different assembly methods and four conspecific strains, the high intraspecific genomic diversity in *L. trichodermophora* was evident in genetic content, potential function and nuclear content.

## supplementary material

10.1099/mgen.0.001218 Uncited Supplementary Material 1.
