# Supplementary material for: Assembly collapsing versus heterozygosity oversizing: detection of homokaryotic and heterokaryotic Laccaria trichodermophora strains by hybrid genome assembly
Source: Microb Genom. 2024 Mar 26;10(3):001218. doi: 10.1099/mgen.0.001218 (PMC10995626; doi:10.1099/mgen.0.001218)

10. Supplementary material

10.1 Supplementary methods

Method 1.

Modifications to the DNeasy Plant Mini Kit (QIAGEN) included: Double amount of fresh filtered mycelial biomass per reaction, addition of a mechanic cell lysis with liquid nitrogen as initial step, increasing the incubation time in the lysis buffer five times, starting the protocols with five reactions for each sample, to finally join the five reactions through a single DNA affinity column, incubating the elution reaction in the DNA affinity column for 5 min before centrifuging and repeating this twice. These modifications were necessary to reach at least 1µg of high integrity DNA as required by sequencing service providers.

Company Limited by Guarantee l Registered in England No. 1039582 l Registered Office Charles Darwin House, 12 Roger Street, WC1N 2JU, UK
Registered as a Charity: 264017 (England & Wales); SC039250 (Scotland)

10.2 Supplementary tables

Table S1. Comparison between *Laccaria trichodermophora* EF-36 ABySS (Short Sequences Assembly) assemblies generated with different *k*-mer sizes.

Assembly	k33	k43	k53	k63	k73
			Contigs		
# Contigs	**4,813**	4,966	5,056	5,186	74
Largest contig	268,483	268,417	407,597	**407,675**	7,885
Total length	31,602,418	35,887,993	38,782,502	**40,934,162**	137,874
GC (%)	48.27	48.15	48.02	47.90	38.66
N50	12,376	14,621	16,272	**16,940**	1,927
L50	**600**	576	570	573	22
# N's per 100 kbp	25.03	**21.08**	27.52	85.18	501.18
			Scaffolds		
# Contigs	**3,633**	3,682	3,759	3,803	49
Largest contig	488,177	332,910	**563,323**	511,709	16,978
Total length	31,641,266	35,928,407	38,827,325	**40,978,089**	138,636
GC (%)	48.27	48.15	48.02	47.90	38.67
N50	18,825	21,647	23,686	**25,468**	4,798
L50	404	**402**	397	406	8
# N's per 100 kbp	155.05	142.03	**140.03**	188.69	968.00

Table S2. Comparison between *Laccaria trichodermophora* EF-36 IDBA_UD (Iterative De Bruijn Assembler with Uneven Depth) assemblies generated with different *k*-mer sizes.

Assembly	k31	k41	k51	k61	k71	k81
# Contigs	6,004	6,326	6,369	6,476	6,437	6,141
Largest contig	108,564	142,015	210,314	303,532	303,552	303,572
Total length	29,141,152	33,409,876	36,644,313	39,142,984	41,079,889	41,947,041
GC (%)	48.31	48.20	48.07	47.91	47.81	47.77
N50	9,231	10,497	12,449	13,714	14,993	16,587
L50	747	740	684	656	631	590
# N's per 100 kbp	0.00	0.00	0.00	0.00	0.00	0.00

	k91	k101	k111	k121	Scaffolds
# Contigs	5,965	5,967	5,837	**5,793**	5,684
Largest contig	407,601	407,621	569,291	**569,530**	569,530
Total length	43,077,763	44,213,521	45,315,083	**46,287,213**	46,287,717
GC (%)	47.71	47.64	47.57	47.51	47.51
N50	18,072	18,991	20,244	**21,378**	21,961
L50	558	537	519	**508**	493
# N's per 100 kbp	0.00	0.00	0.00	0.00	0.05

Table S3. Comparison between *Laccaria trichodermophora* EF-36 SPAdes assemblies generated with a different set of *k*-mer sizes.

Assembly	Default	Step10	Step5	Default	Step10	Step5
		Contigs			Scaffolds	
# Contigs	**3,915**	5,034	5,331	**3,785**	4,881	4,701
Largest contig	**504,919**	447,787	478,617	**504,919**	447,787	478,617
Total length	43,812,471	44,868,831	**47,293,303**	43,837,338	44,965,206	**48,054,544**
GC (%)	47.70	47.61	47.48	47.70	47.61	47.43
N50	**28,693**	23,414	23,761	**29,857**	24,320	28,092
L50	**372**	455	478	**362**	442	419
# N's per 100 kbp	0.00	0.00	0.00	29.06	**21.92**	27.75

Table S4. Comparison between *Laccaria trichodermophora* EF-36 Velvet assemblies generated with different *k*-mer sizes.

Assembly	k63	k65	k67	k69	k71
**Contigs**	7,058	8,006	**11,717**	13,394	156
**Largest contig**	**177,176**	94,476	25,735	14,069	26,051
**Total length**	38,634,369	**38,636,637**	36,990,772	21,716,930	285,257
**GC (%)**	47.92	47.91	47.92	48.03	41.86
**N50**	**10,791**	8,460	4,116	1,632	1,628
**L50**	**862**	1,192	2,699	4,763	39
**# N's per 100 kbp**	0.00	0.00	0.00	0.00	0.00

Table S5. Comparison between *Laccaria trichodermophora* EF-36 best results of different short-reads genome assemblers.

Assembly	ABySS		IDBA_UD	
	Contigs	Scaffolds	Contigs	Scaffolds
**Contigs**	5,186	3,803	5,793	5,684
**Largest contig**	407,675	511,709	**569,530**	**569,530**
**Total length**	40,934,162	40,978,089	**46,287,213**	**46,287,717**
**GC (%)**	47.90	47.90	47.51	47.51
**N50**	16,940	25,468	21,378	21,961
**L50**	573	406	508	493
**# N's per 100 kbp**	85.18	188.69	0.00	0.05
	**SPAdes**		**Velvet**	
	**Contigs**	**Scaffolds**	**Contigs**	**Scaffolds**
**Contigs**	**3,915**	**3,785**	7,058	
**Largest contig**	504,919	504,919	177,176	-
**Total length**	43,812,471	43,837,338	38,634,369	-
**GC (%)**	47.70	47.70	47.92	-
**N50**	**28,693**	**29,857**	10,791	-
**L50**	**372**	**362**	862	-
**# N's per 100 kbp**	0.00	29.06	0.00	-

Table S6. Long reads and hybrid assemblies' comparison.

Assembly	Hyb-SPADes	MaSuRCA	Wtdbg2	Fly	Canu (smash)	Canu	Pilon	PurgeHap
**Strain**					**11Lt**			
**Contigs**	9,346	1,422	925	1,744	2,280	1,119	**886**	**876**
**Largest contig (Kbp)**	140.61	1,352.51	**1,502.98**	1,062.65	1,174.68	917.15	1,052.63	1,052.63
**Total length (Mbp)**	85.23	**115.56**	74.55	96.13	126.57	111.69	112.18	111.90
**GC (%)**	47.15	46.44	46.38	46.57	46.60	46.65	46.66	46.67
**N50 (Kbp)**	20.48	140.74	207.33	106.93	101.82	171.95	**238.47**	241.04
**L50**	1,133	216	**91**	**234**	309	180	134	133
**# N's per 100 kbp**	4,574.74	1.56	**0.00**	1.46	**0.00**	**0.00**	348.50	349.37
**Strain**					**36Lt**			
**Contigs**	1,363	241	224	144	240	94	**77**	**77**
**Largest contig (Kbp)**	1,506.71	3,140.83	2,571.08	2,449.43	2,237.28	4,959.16	**5,316.68**	**5,316.68**
**Total length (Mbp)**	53.20	**61.05**	52.44	57.78	60.68	58.99	59.06	59.06
**GC (%)**	47.02	46.73	46.89	46.87	46.83	46.84	46.84	46.84
**N50 (Kbp)**	158.18	**624.15**	522.86	976.43	943.56	1,351.60	1,464.17	1,464.17
**L50**	86	27	24	17	**21**	**12**	**12**	**12**
**# N's per 100 kbp**	123.28	0.33	**0.00**	0.52	**0.00**	**0.00**	91.36	91.36

All values are considering contigs/scaffolds over 1Kb; Fly, Canu and Canu smash assemblies were made using Long-Reads, wile the remaining five with a hybrid approach: Pilon were made using the Canu results and PurgeHap using the Pilon results.

Table S7. MaxBin analysis results.

	Abundance %	Coverage %	Genome size Mpb
		**36.LR**	
Bin 1	0.12	1.9	21
Bin 2	0.12	11.2	37
		**11.LR**	
Bin 1	0.09	3.7	48
Bin 2	0.08	13.1	65

Table S8. *Laccaria trichodermophora* genomic sequences BLAST matching the HD1 mating-type protein (a1-1) (XM_001873350.1) of *L. bicolor*.

Description	Max Score	Total score	Query coverage	E value	Percent identity	Accession
Laccaria trichodermophora strain CA15-F10 10Lt_node_1787, whole genome shotgun sequence	300	300	89%	4e-87	34.91%	JACTVE01000 1787.1
Laccaria trichodermophora strain CA15-75 75Lt_node_2275, whole genome shotgun sequence	233	316	80%	1e-78	43.20%	JACTVB01000 2275.1
Laccaria trichodermophora strain EF-36 36Lt_node_10, whole genome shotgun sequence	192	302	52%	2e-74	49.07%	JACTVC01000 0010.1
Laccaria trichodermophora strain CA15-F10 10Lt_node_1540, whole genome shotgun sequence	249	358	65%	1e-69	46.69%	JACTVE01000 1540.1
Laccaria trichodermophora strain CA15-75 75Lt_node_315, whole genome shotgun sequence	193	321	47%	6e-51	52.49%	JACTVB01000 0315.1
Laccaria trichodermophora strain CA15-11 11Lt_node_718, whole genome shotgun sequence	193	307	64%	1e-50	39.45%	JACTVD01000 0718.1
Laccaria trichodermophora strain CA15-11 11Lt_node_15, whole genome shotgun sequence	193	307	64%	1e-50	39.45%	JACTVD01000 0015.1

10.3 Supplementary figures

Figure S1. The classical *Laccaria* life cycle. Tetrasporic basidia produces four monokaryotic and haploid basidiospores (n). Basidiospores produces primary mycelia (n). Two compatible primary mycelium fuse by plasmogamy (P!) to form dikaryotic heterokaryotic secondary mycelia (n + n). Secondary mycelia perform most physio-ecological activities such as vegetative growth, soil exploration, ectomycorrhizal symbiosis, and basidiomata production. In the basidiomata gills, the immature basidia nuclei fuse by karyogamy (K!) forming a diploid nucleus (2n). Meiosis (M!) divides the genomic content of the dikaryotic nucleus and post-meiotic mitoses multiply into four haploid nuclei. New recombinant haploid nuclei migrate to basidiospores for dispersion (mid gray). Background pizza-plot represents: the ephemeral Diploid stage (dark gray), the short-lived haploid stage (mid gray), and the dominant dikaryotic stage (light gray).

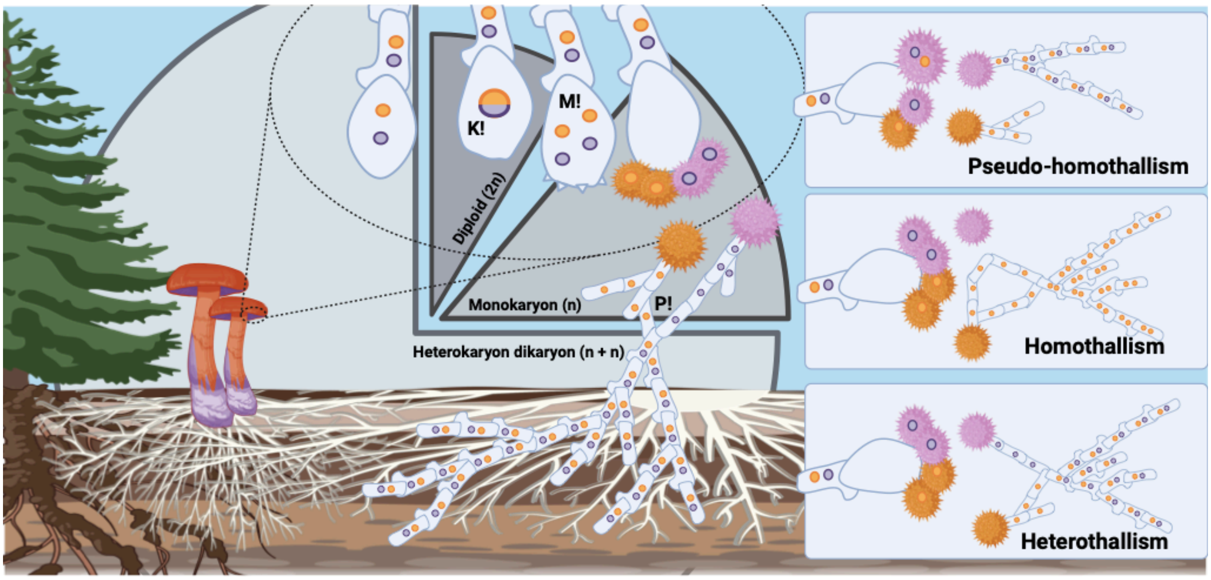

Figure S2. Quality control of raw and clean Next-Seq (76b) pair end reads of CA15-11 strain.

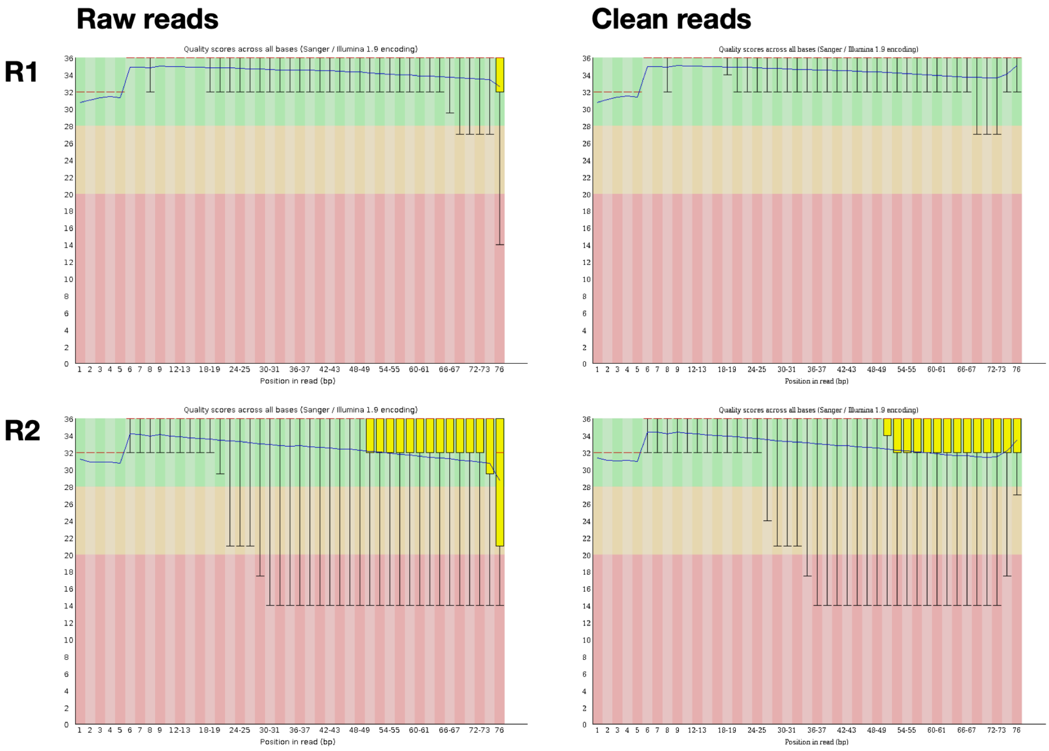

Figure S3. Quality control of raw and clean Mi-Seq (300b) pair end reads of CA15-11 strain.

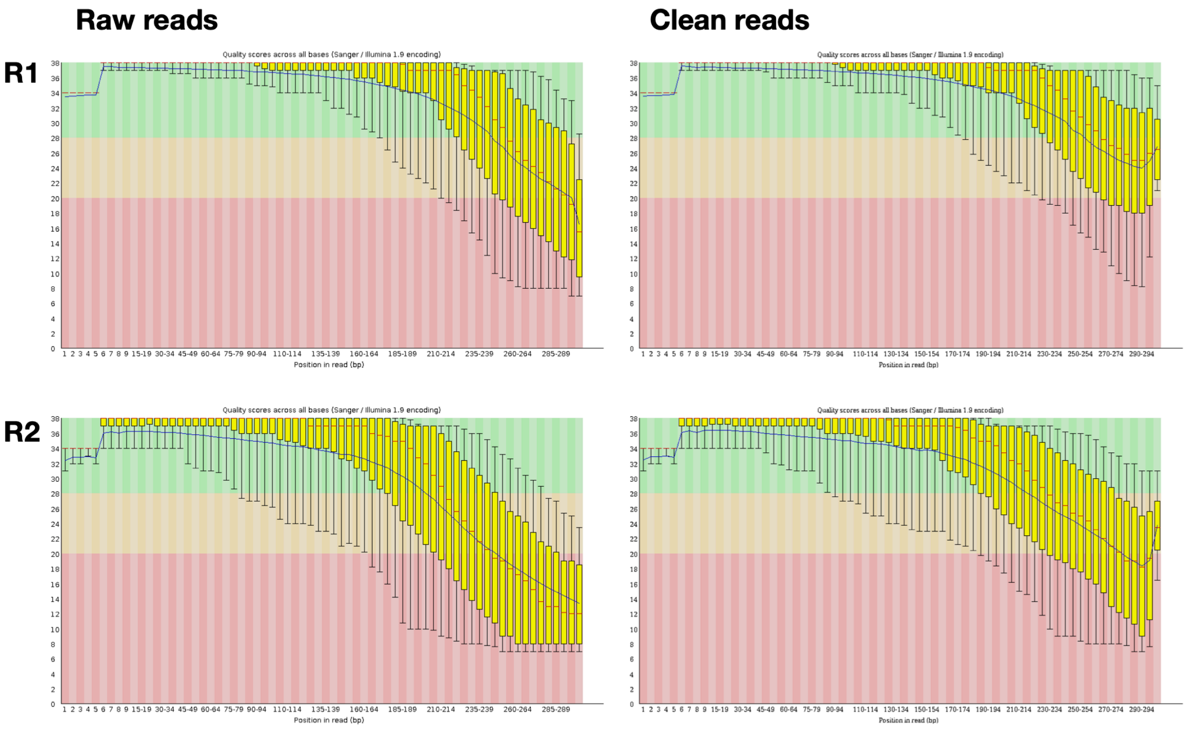

Figure S4. Quality control of raw and clean Next-Seq (76b) pair end reads of CA15-75 strain.

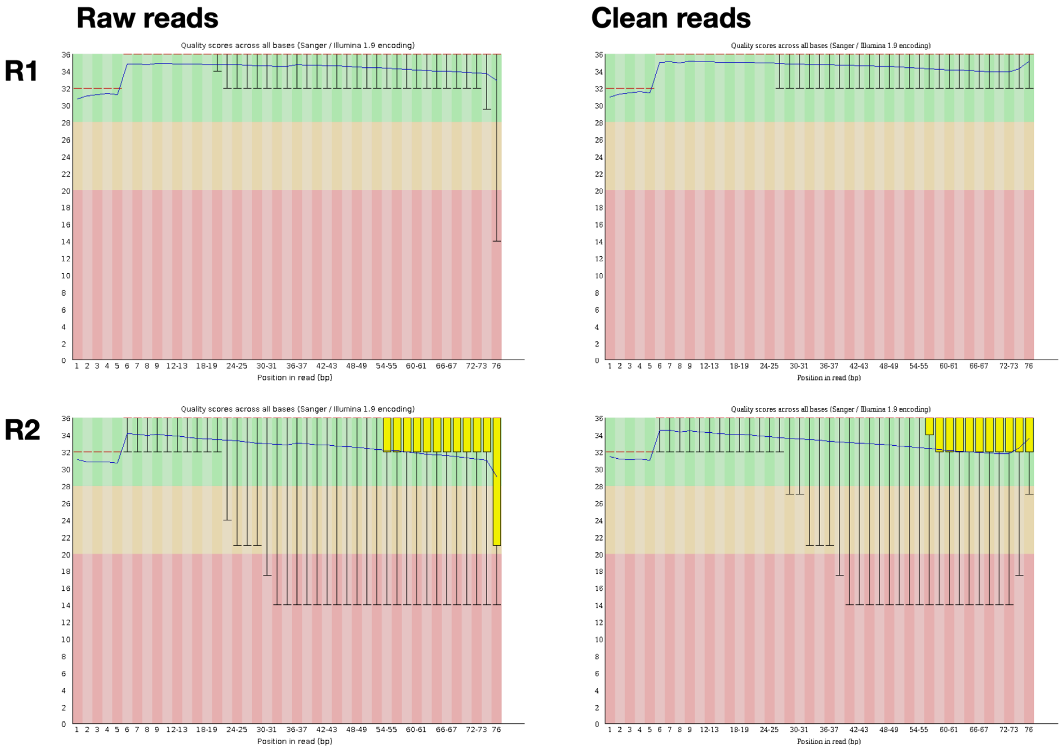

Figure S5. Quality control of raw and clean Mi-Seq (300b) pair end reads of CA15-75 strain.

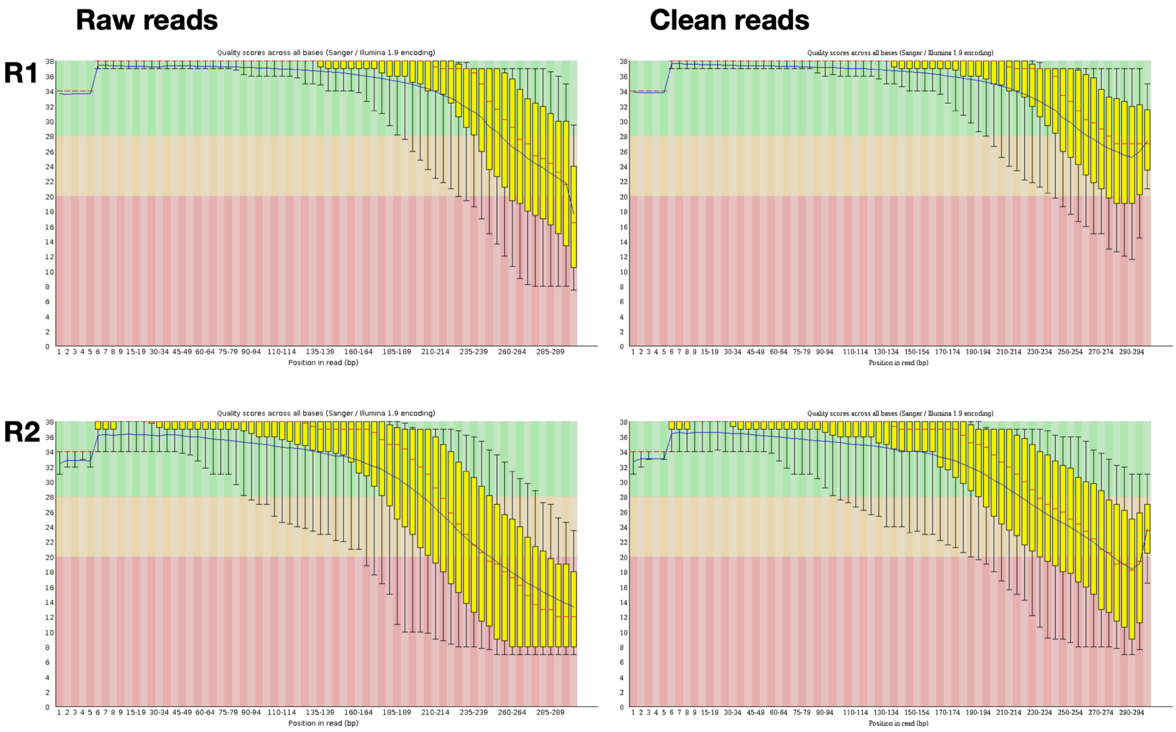

Figure S6. Quality control of raw and clean Next-Seq (76b) pair end reads of CA15-F10 strain.

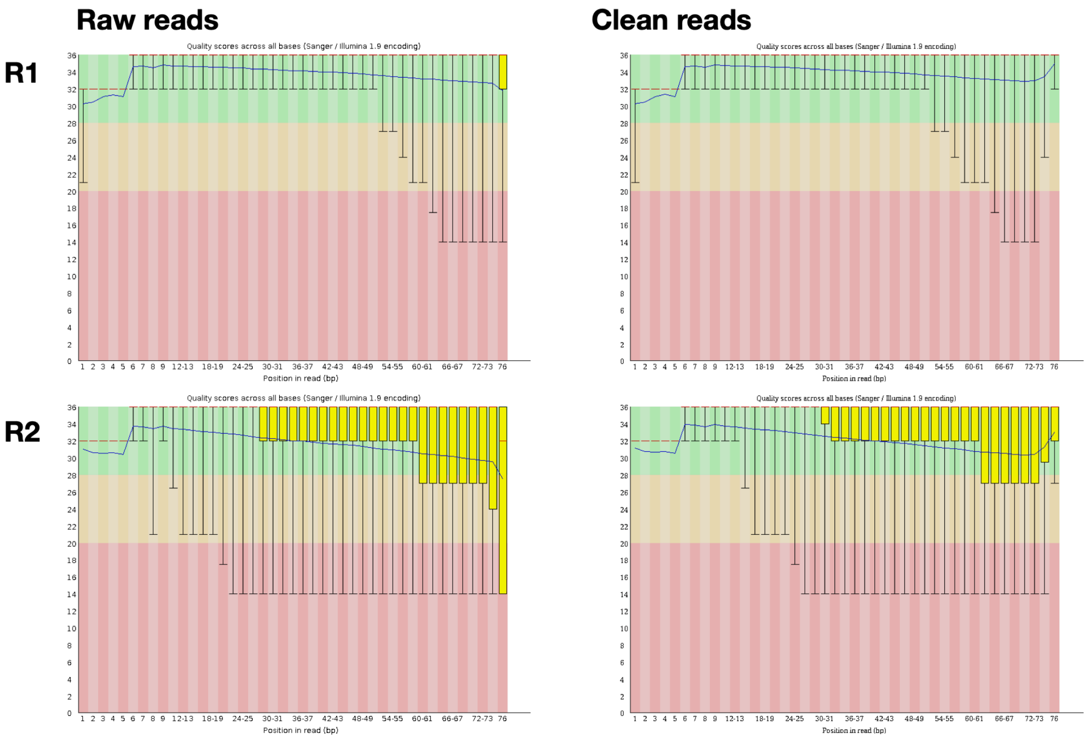

Figure S7. Quality control of raw and clean Mi-Seq (300b) pair end reads of CA15-F10 strain.

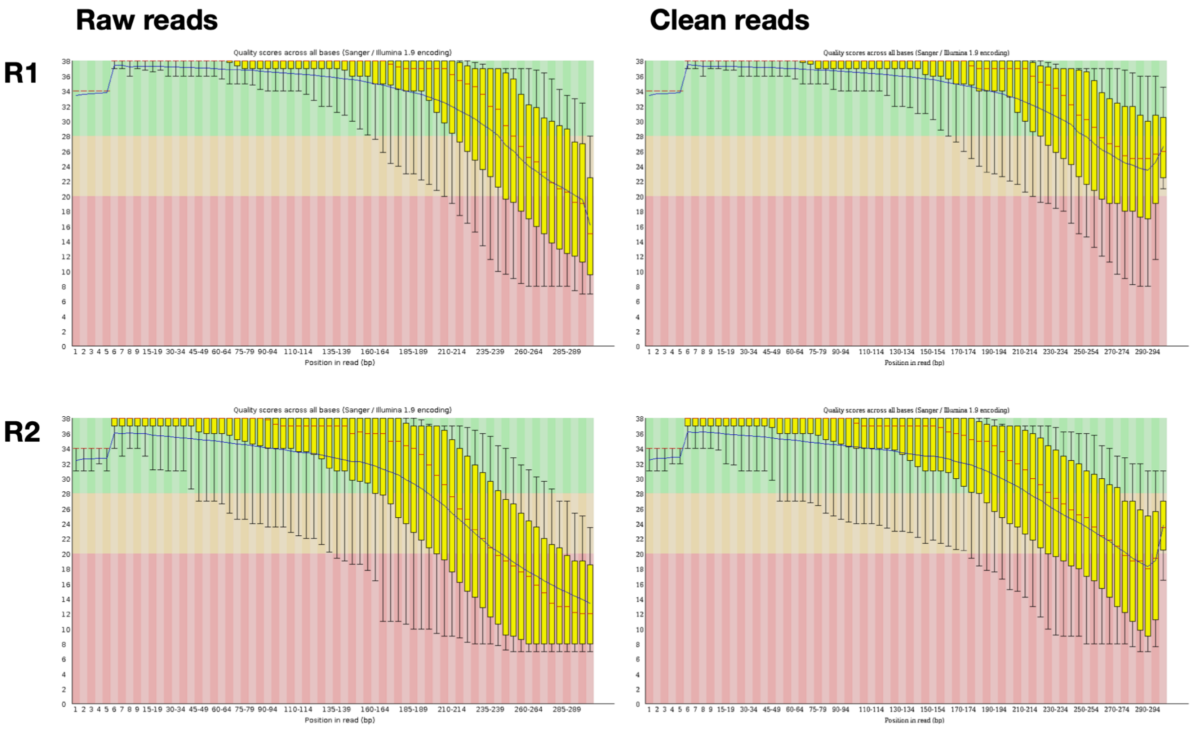

Figure S8. Quality control of raw and clean Next-Seq (76b) pair end reads of EF-36 strain.

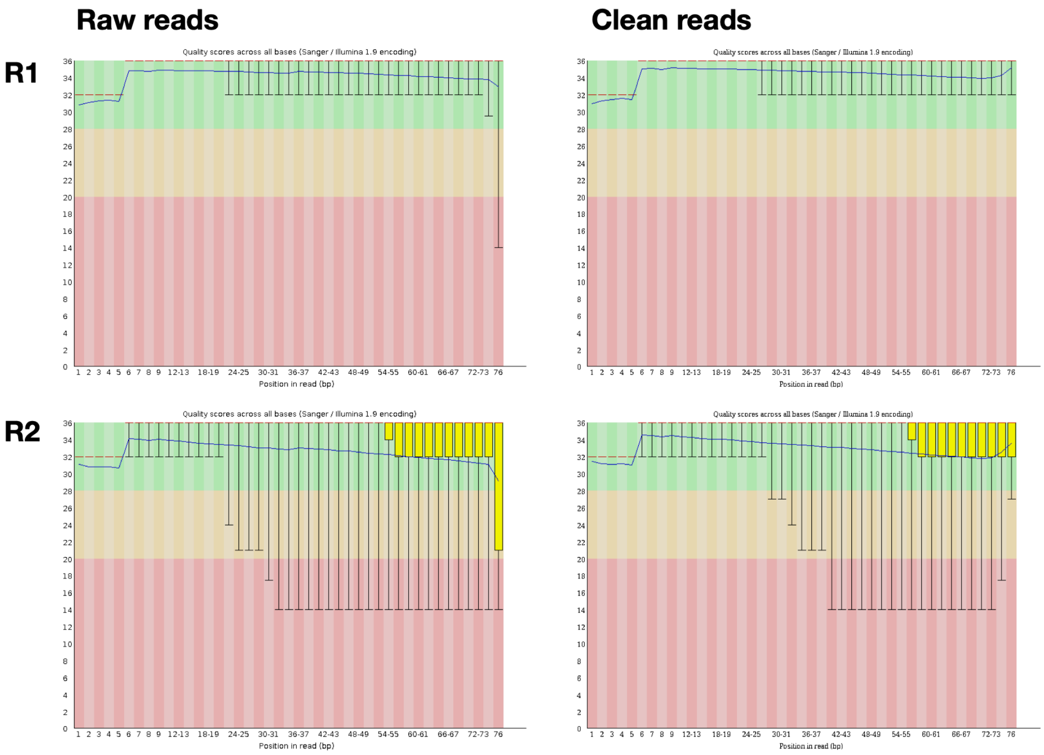

Figure S9. Quality control of raw and clean Mi-Seq (300b) pair end reads of EF-36 strain.

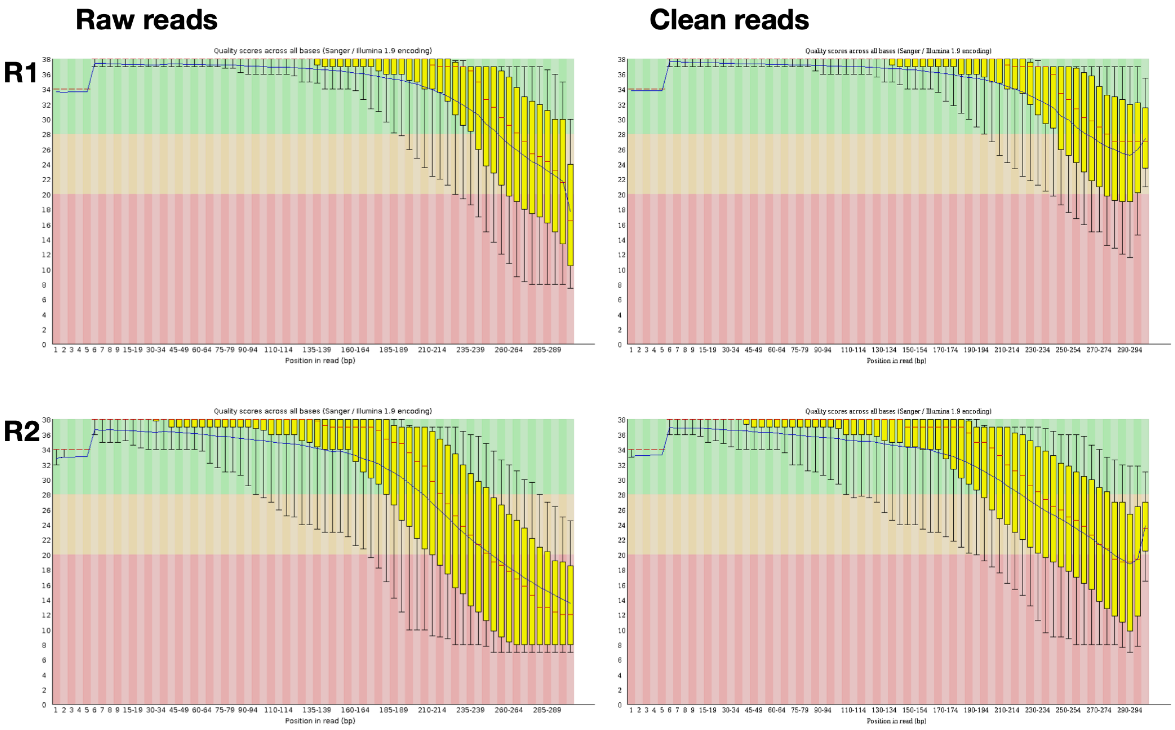

Figure S10. General workflow for the whole de novo *Laccaria trichodermophora* genome assembly. **A)** Short read assemblies, **B)** Long read of hybrid assemblies. Lilac boxes represent sequencing raw, trimmed or corrected data, low blue boxes represent assemblers, yellow boxes represent complementary software such as cleaner, mapping or polishing tools, green boxes represent resulting assemblies.

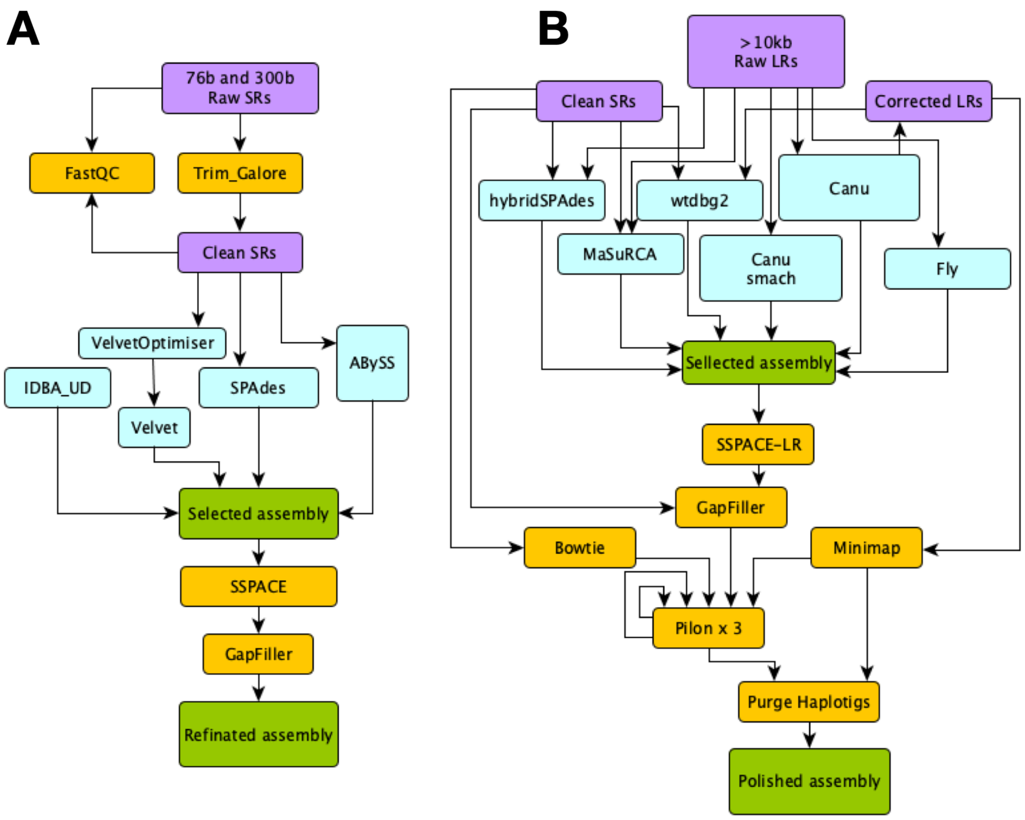

Figure S11. Correlation between read length (x) and genome assembly sizes (y) of *Laccaria trichodermophora*.

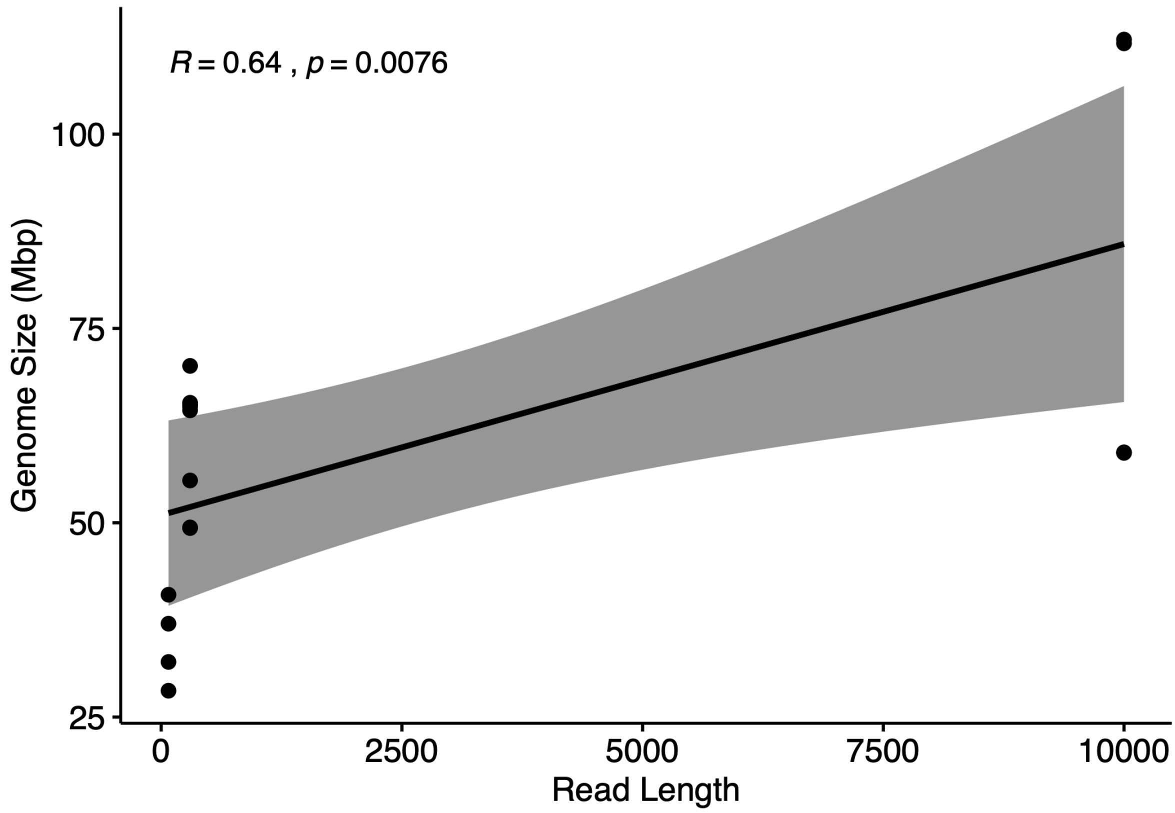

Figure S12. Icarus alignment viewer of *L. trichodermophora* CA15-11 assemblies generated with Quast using the CA15-11 hybrid (Canu + Pilon) assembly as reference. **A)** Display a fragment of the alignment in B. **B)** display first 177 scaffolds.

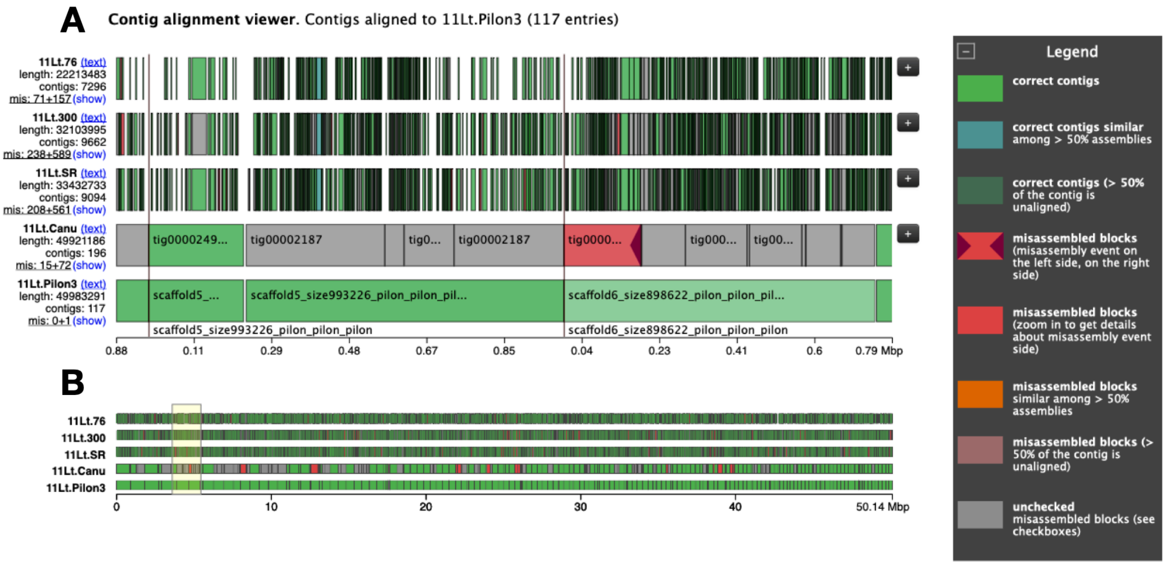

Figure S13. Icarus alignment viewer of *L. trichodermophora* EF-36 assemblies generated with Quast using the EF-36 hybrid (Canu + Pilon) assembly as reference. **A)** Display a fragment of the alignment in B. **B)** display first 31 scaffolds.

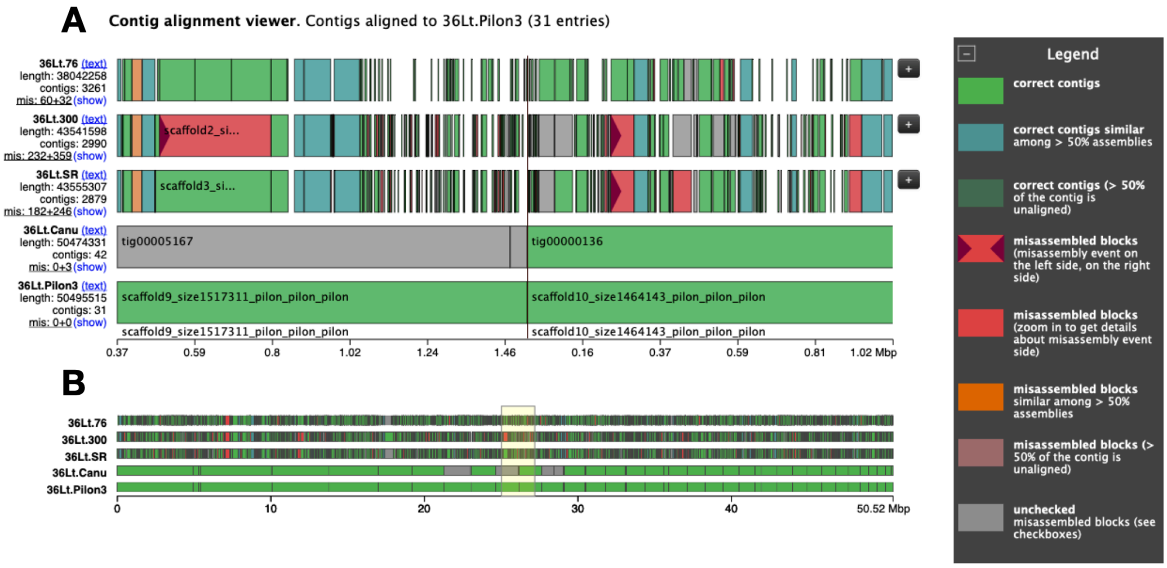

Figure S14. Vizbin v1.0.0 scatterplot of the contigs of the hybrid assemblies of *Laccaria trichodermophora* strains CA15-11 and EF-36. Each blue dot corresponds with one contig.

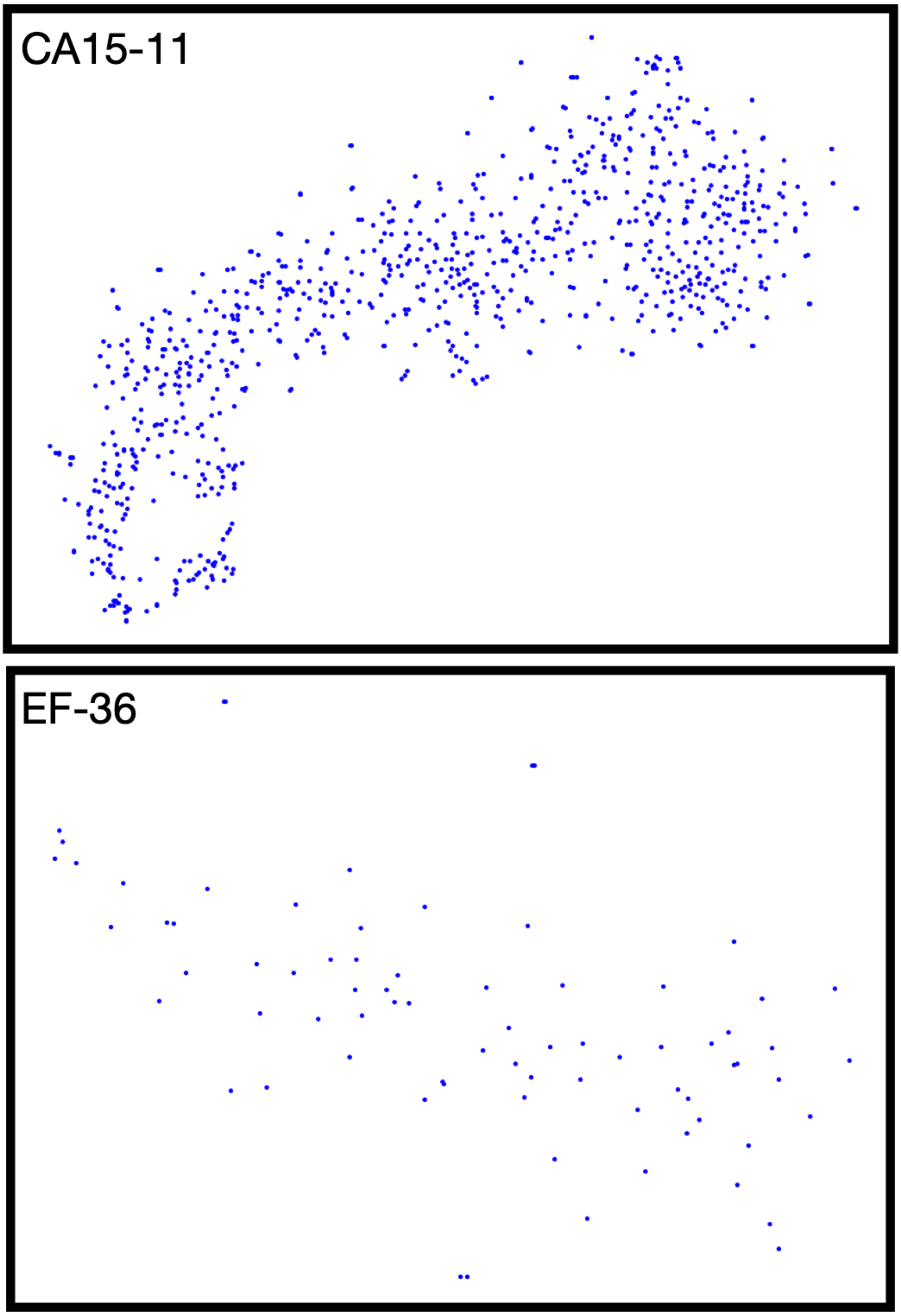

Figure S15. Jelifish-Genome Scope analysis of the three heterokaryons (CA5-11, CA15-75 and CA15-F10) and one homokaryon (EF-36) *L. trichodermophora* strains. Heterokaryotic strains display a two-pick curve while monokaryon display a single central one. *K*-mer estimations of genome size, coverage, duplication and heterozygosity are also presented.

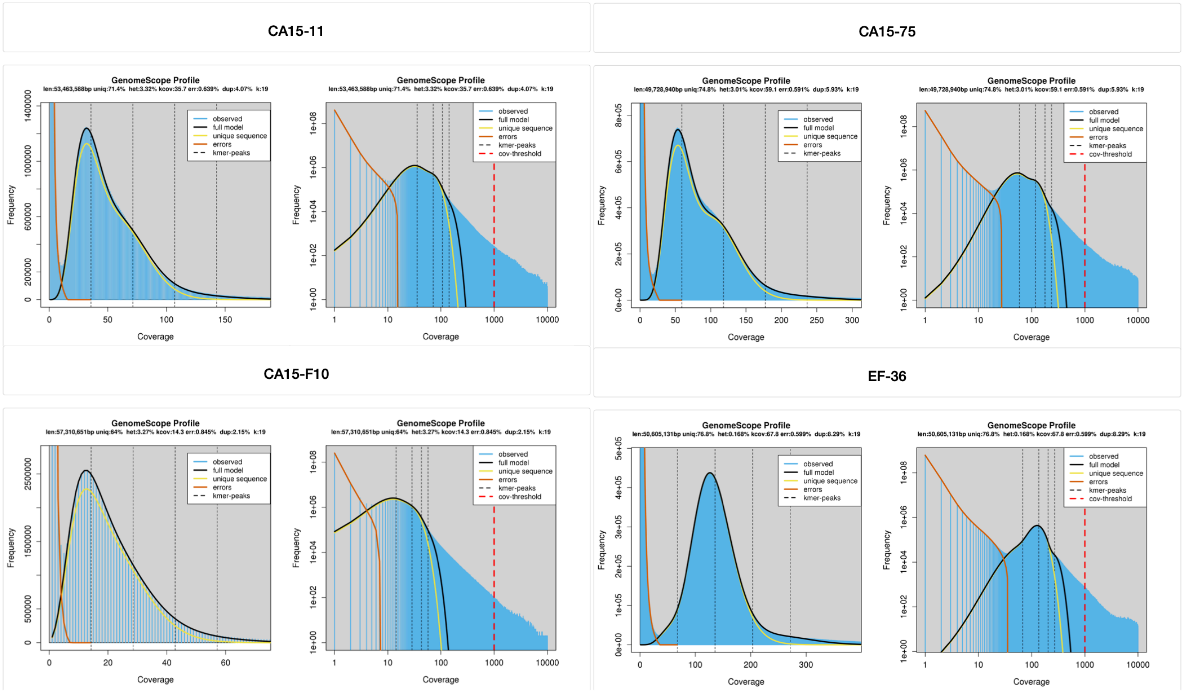

Figure S16. Blast matches of the homeodomain transcription factor, HD1 mating-type protein (a1-1) (XM_001873350.1) of *L. bicolor* on the *L. trichodermophora* genomes.

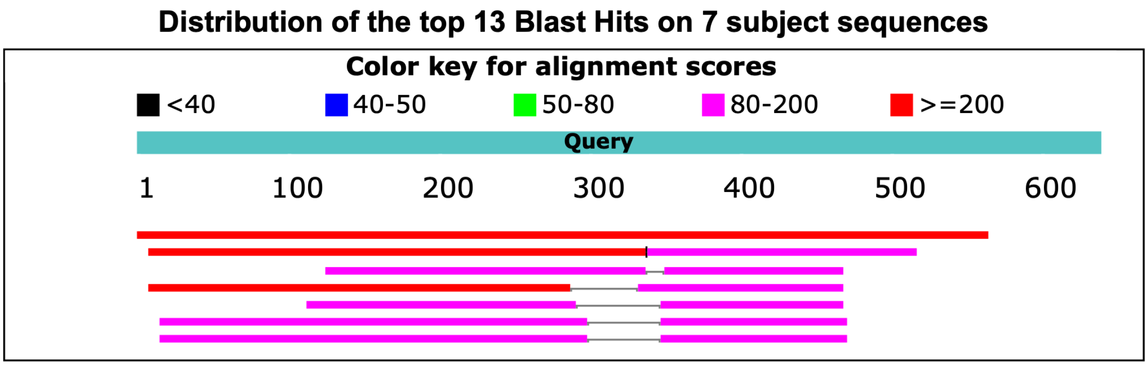

Figure S17. Blast searches of the MAT-A region (query) vs *L. trichodermophora* genomes (subject).

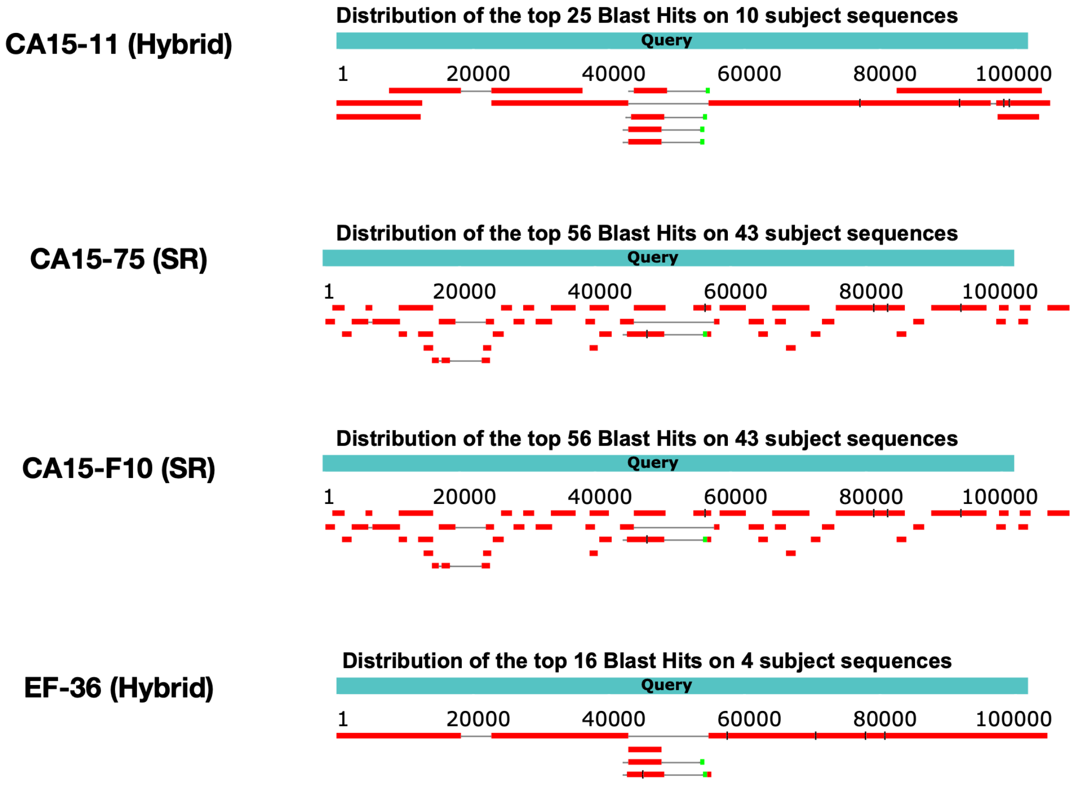

Figure S18. Collinearity analysis (mummer) between the contigs of two *L. trichodermophora* strains (CA15-11 and EF-36) containing the genes present in the MAT-A region of *L. bicolor*. It is observed that the contigs of the CA15-11 strain align twice against those of the EF-36 strain, except for the region that goes from 40,000 to 60,000 of EF-36. Red colors represent greater nucleotide identity than blue colors.

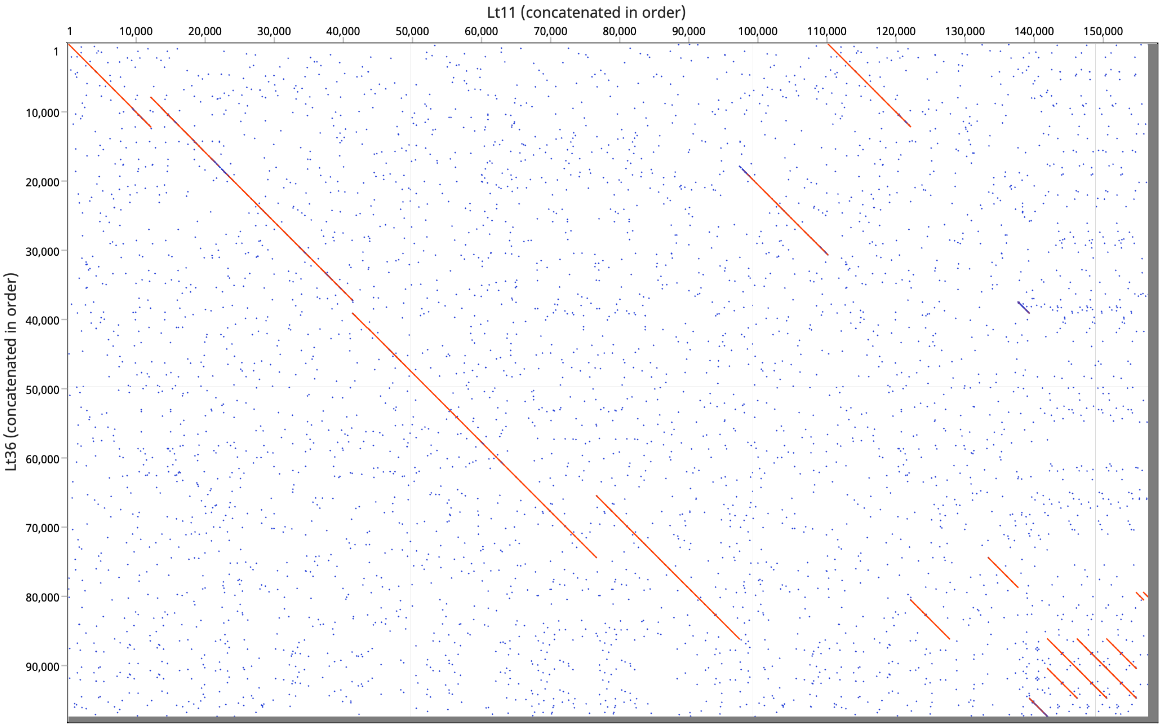

Figure S19. CAZyme genes gain and lose in four different strains/assemblies.

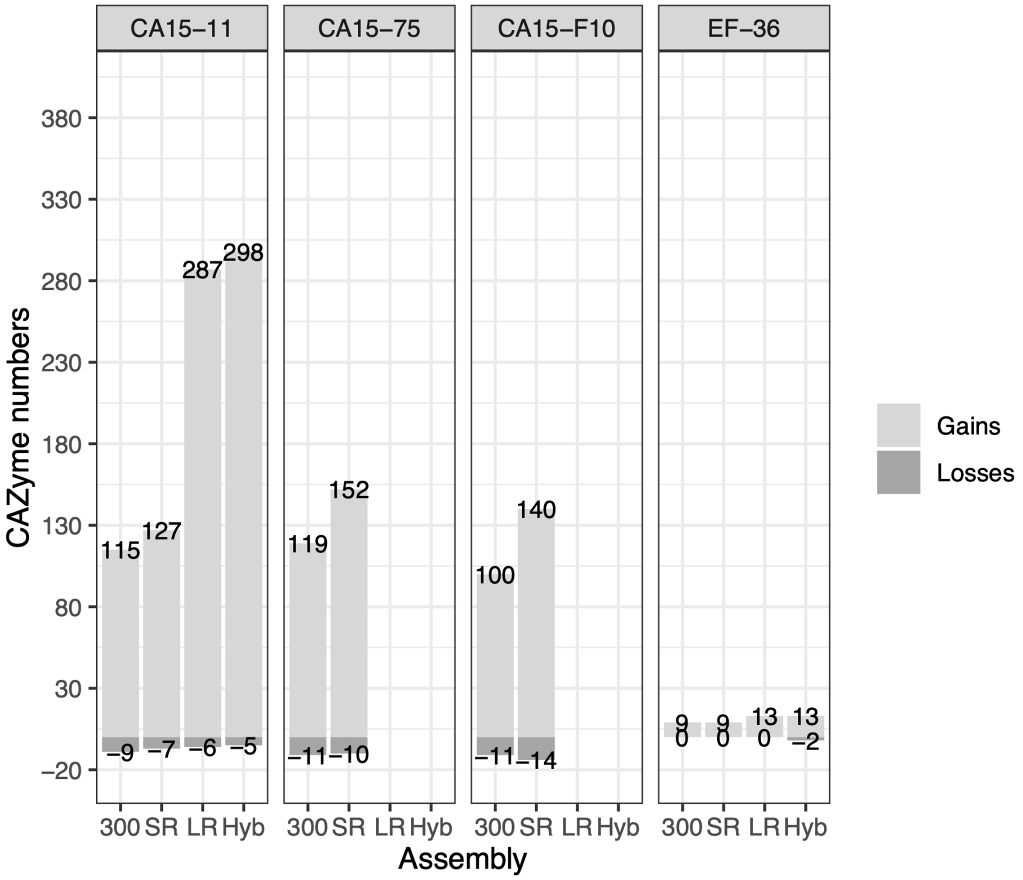

Figure S20. Heatmap comparing the predicted CAZy gene content. Row dendrogram clusters together enzymes with similar copy number means.

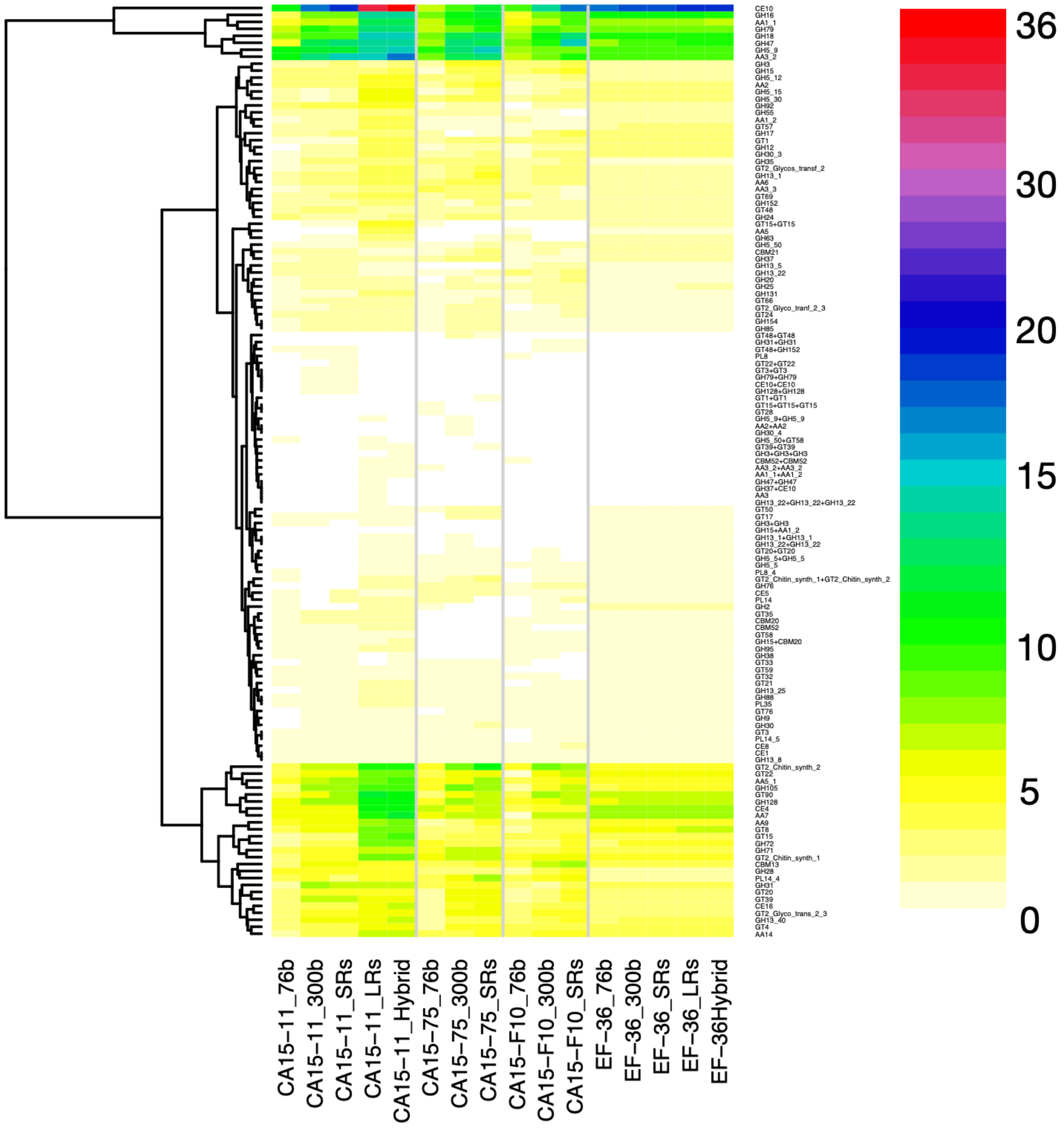

Figure S21. Microscopic features of two *L. trichodermophora* strains and its provenance basidiomata. Left: CA15-11 (Heterokaryon); Right EF-36 (Homokaryon). A, B, E, F: Bisporic or trisporic basidia; C, D, G, H: Fibulated hyphae from the basidiomata context.

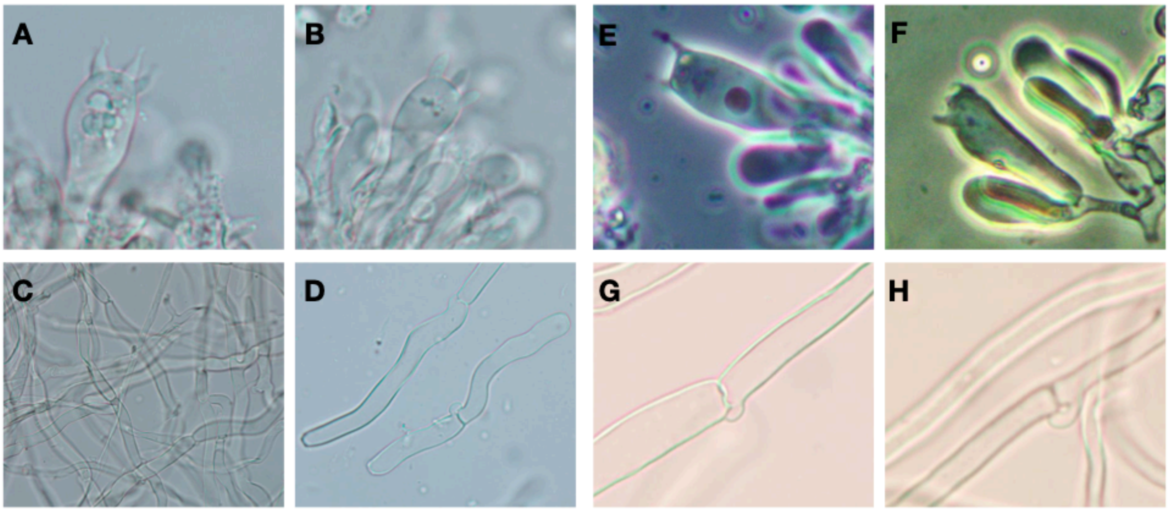

Supplement: Uncited Supplementary Material 1. [file mgen-10-01218-s001.pdf]
